# Supplementary material for: Nitrogen Enriched Solid-State Cultivation for the Overproduction of Azaphilone Red Pigments by Penicillium sclerotiorum SNB-CN111
Source: J Fungi (Basel). 2023 Jan 24;9(2):156. doi: 10.3390/jof9020156 (PMC9958536; doi:10.3390/jof9020156)
Supplement: Supplementary file 1 [file jof-09-00156-s001.zip › jof-2163988-supplementary.pdf]

Nitrogen enriched solid-state cultivation for the overproduction of azaphilone red pigments by *Penicillium sclerotiorum* SNB-CN111

Téo Hebra[1], Véronique Eparvier[1*], David Touboul[1,2*]

[1] Université Paris-Saclay, CNRS, Institut de Chimie des Substances Naturelles, UPR 2301, 91198, Gif-sur-Yvette, France.

[2] LCM, CNRS, Ecole polytechnique, Institut Polytechnique de Paris, Route de Saclay, 91120 Palaiseau, France.

Figure S1: Spectral comparison with databases of cluster A annotated molecules with their theoretical protonated *m/z*. 4
Figure S2: Spectral comparison with databases of cluster B annotated molecules with their theoretical protonated *m/z*. 5
Figure S3: Spectral comparison of cluster C molecules with databases with their theoretical protonated *m/z*. 6
Figure S4: Spectral comparison of cluster D molecules with databases and their theoretical protonated *m/z*. 8
Figure S5: Spectral comparison of cluster E molecules with databases and their theoretical protonated *m/z*. 9
Figure S6: Subdivision of Cluster D based on annotated features 12
Figure S7: Annotation of cluster E with hypothetical chain structures integrated to the azaphilone scaffold, proposed from molecular network data and KEGG compound database [12]. epoxy means The 3,5-dimethyl-1,3-heptadienyl unit bear an epoxide on position 3. Cl means azaphilone is chlorinated 13
Figure S8: HRMS spectrum for compound **24** 14
Figure S9: ^{1}H NMR spectrum (CD_3CN) of compound **24** 15
Figure S10: ^{13}C NMR spectrum (CD_3CN) of compound **24** 16
Figure S 11: HRMS spectrum for compound **39** 17
Figure S12: ^{1}H NMR spectrum ($CDCl_3$) of compound **39** 18
Figure S13: ^{13}C NMR spectrum ($CDCl_3$) of compound **39** 19
Figure S14: HRMS spectrum for compound **50** 20
Figure S15: ^{1}H NMR spectrum (MeOD) of compound **50** 21
Figure S16: ^{13}C NMR spectrum (MeOD) of compound **50** 22
Figure S17: Comparison of fragmentation spectra and retention time from hemisynthetized standards with metabolites **24**, **39** and **50** produced by *P. sclerotiorum* SNB-CN111. 23
Figure S18*:* Extracted chromatogram for molecules **1** ($[M+H]^+$, *m/z* 391.1307), **3** ($[M+H]^+$, *m/z* 390.1467) and **4** ($[M+H]^+$, *m/z* 434.1729) as a function of the concentration of ammonium acetate or ethanolamine added to solid CZK medium. 25
Figure S19: Metabolic profile of from *P. sclerotiorum* SNB-CN111 fermentation on solid CZK crude extract and two step fermentation process on porous cellophane membrane on solid CZK supplemented with propargilamine 0.5 $g.L^{-1}$. 26
Figure S20: Base pic chromatogram of fraction F1 from flash chromatography of pulled two step fermentation process on porous cellophane membrane on solid CZK supplemented with propargilamine crude extracts. 27
Figure S21 HRMS of compound **63**. 27
Figure S22: ^{1}H NMR spectrum ($CDCl_3$) of compound **63** 28
Figure S23: ^{13}C NMR spectrum ($CDCl_3$) of compound **63**. 29
Figure S 24: COSY NMR spectrum ($CDCl_3$) of compound **63** 30
Figure S 25: HSQC NMR spectrum ($CDCl_3$) of compound **63**. 31
Figure S 26 HMBC NMR spectrum ($CDCl_3$) of compound **63**. 32
Figure S27: Key ^{1}H–^{1}H COSY (bold bonds) and ^{1}H–^{13}C (plain arrow) correlations of compound **63.** 33

Table S1: Cluster A features annotated by MS/MS database query. In-house database was built upon annotations from Hebra et al.[1] 3
Table S2: Cluster B features annotated by MS/MS database query. In-house database was built upon annotations from Hebra et al.[1] 5
Table S3: Cluster C features annotated by MS/MS database query. In-house database was built upon annotations from Hebra et al.[1] 6

Table S4 : Cluster D features annotated by MS/MS database query. In-house database was built upon annotations from Hebra et al.[1] ... 7
Table S5: Cluster E features annotated by MS/MS database query. In-house database was built upon annotations from Hebra et al.[1] ... 9
Table S6: Annotation of azaphilones from cluster D-I with proposed chain structure from molecular network data and KEGG compound database.[12] ... 10
Table S7: Annotation of azaphilones from cluster D-II with proposed chain structure from molecular network data and KEGG compound database.[12] ... 11
Table S8: Annotation of azaphilones from cluster D-III with proposed chain structure from molecular network data and KEGG compound database.[12] ... 11
Table S9: Annotation of azaphilones from cluster E with proposed chain structure from molecular network data and KEGG compound database.[12] ... 12
Table S10: Absorbance at 500, 460 and 400 nm of *P. sclerotiorum* SNB-CN111 crude extracts from fermentation on solid CZK, PD and YE. Values are given in AU.g^{-1} of culture medium. ... 23
Table S11: Absorbance at 500, 460 and 400 nm of *P. sclerotiorum* SNB-CN111 crude extracts from fermentation on solid CZK supplemented with ammonium acetate or ethanolamine at 0, 0.1, 0.5 and 2 g.L^{-1}. Values are given in AU.g^{-1} of culture medium ... 24
Table S12: Absorbance at 500, 460 and 400 nm of *P. sclerotiorum* SNB-CN111 crude extracts from two step fermentation process on porous cellophane membrane on solid CZK supplemented with ammonium acetate or ethanolamine at 0, 0.1, 0.5 and 2 g.L^{-1}. Values are given in AU.g^{-1} of culture medium. ... 25
Table S13: Absorbance at 500, 460, and 400 nm of *P. sclerotiorum* SNB-CN111 crude extracts from two step fermentation process on porous cellophane membrane on solid CZK supplemented with propargilamine at 0, 0.1, 0.5 and 2 g.L^{-1}. Values are given in AU.g^{-1} of culture medium. ... 25
Table S14: ^{1}H NMR spectroscopic data for compounds **3**, **4**, **39′** and **63** recorded at 500 MHz on $CDCl_3$, **24′** recorded at 500 MHz on CD_3CN and **50′** recorded at 500 MHz on MeOD. Compounds **3** and **4** were described on a previous study.[1] ... 34
Table S15: ^{13}C NMR spectroscopic data for compounds **3**, **4**, **39′** and **63** recorded at 500 MHz on $CDCl_3$, **24′** recorded at 700 MHz on CD_3CN and **50′** recorded at 500 MHz on MeOD. Compounds **3** and **4** were described on a previous study.[1] ... 35

Table S1: Cluster A features annotated by MS/MS database query. In-house database was built upon annotations from Hebra et al.[1]

ClusterA									
$[M+H]^+$	Error (ppm)	Formula	Name	Database	Cosine score	Annotation	Ref	$\varepsilon(\lambda_{nm})$	Minimal condition
357.1706	-2.7	$C_{21}H_{24}O_5$		In-house	0.97	Level 2	[1,2]		CZK
359.1863	-2.8	$C_{21}H_{26}O_5$		In-house	0.72	Level 3	[1]		PD
363.1349	-2.4	$C_{20}H_{23}ClO_4$		In-house	0.88	Level 2	[1]		CZK
371.1859	-1.6	$C_{22}H_{26}O_5$		In-house	0.85	Level 2	[1]		PD
385.2013	-0.9	$C_{23}H_{28}O_5$		In-house	0.86	Level 3	[1]		PD
391.1315	-2.1	$C_{21}H_{23}ClO_5$	Sclerotiorin	MS-Dial	0.84	Level 1	[3,4]	12000(445)	CZK
395.1633	-3.4	$C_{21}H_{27}ClO_5$	Isochromophilone IV	MS-Dial	0.66	Level 2	[5]	39400(390)	CZK
399.2177	-2.8	$C_{24}H_{30}O_5$		In-house	0.9	Level 3	[1]		PD
405.1473	-2.4	$C_{22}H_{25}ClO_5$	Sclerketide B	In-house	0.95	Level 2	[6]	17(332)	PD
407.1261	-1.3	$C_{21}H_{23}ClO_6$		In-house	0.73	Level 3	[1]		PD
419.163	-2.4	$C_{23}H_{27}ClO_5$		In-house	0.93	Level 3	[1]		PD
433.1785	-2.0	$C_{24}H_{29}ClO_5$		In-house	0.7	Level 3	[1]		PD
435.1578	-2.1	$C_{23}H_{27}ClO_6$		In-house	0.63	Level 3	[1]		PD

Level 0 corresponds annotations from isolated pure compounds; level 1 to annotations by comparison with standards; level 2 to putative annotations (e.g., MS/MS library comparison or tentative structure); level 3 to a chemical class assignment

Figure S1: Spectral comparison with databases of cluster A annotated molecules with their theoretical protonated *m/z*.

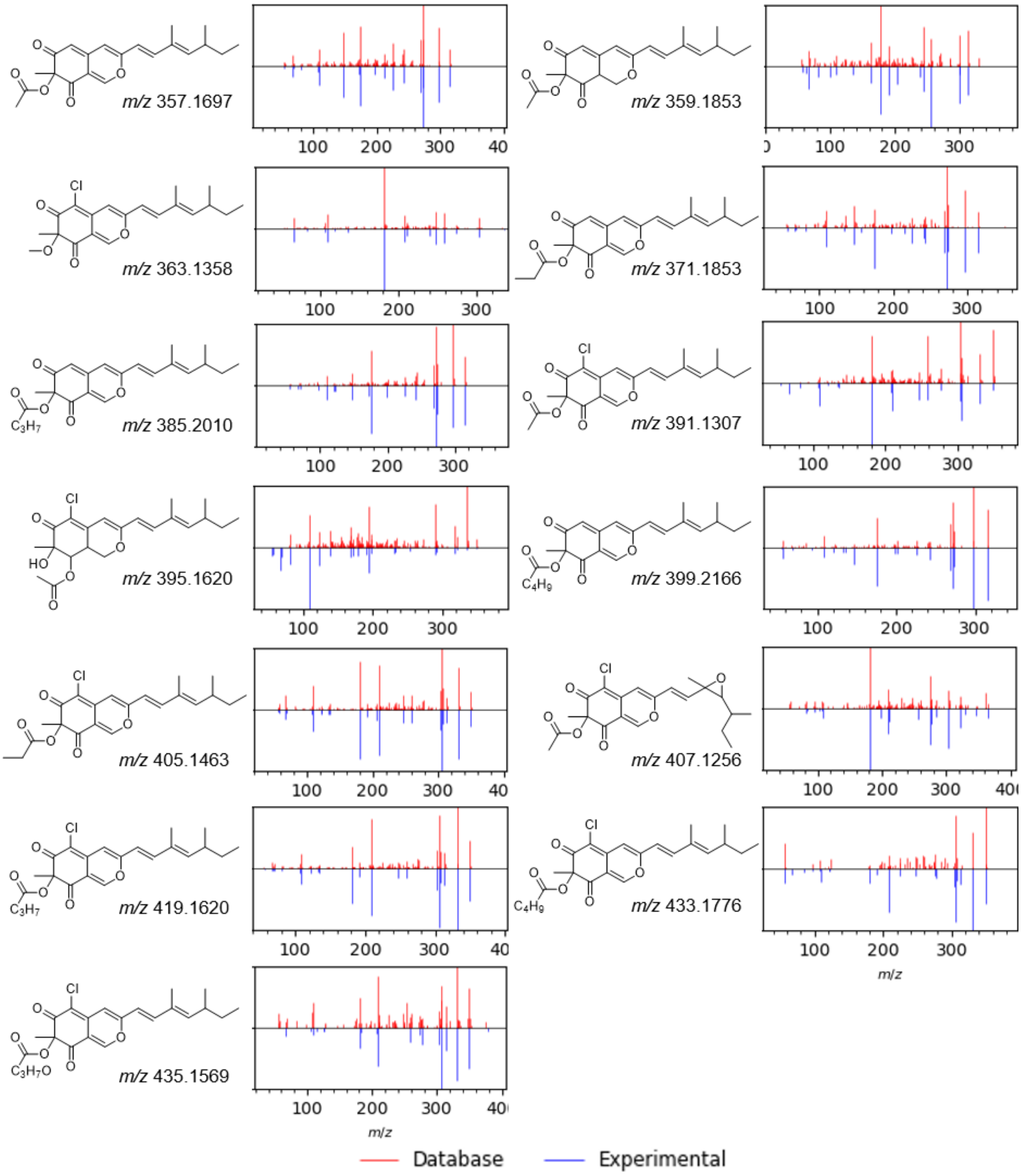

Table S2: Cluster B features annotated by MS/MS database query. In-house database was built upon annotations from Hebra et al.[1]

ClusterB									
$[M+H]^+$	Error (ppm)	Formula	Name	Database	Cosine score	Annotation	Ref	$\varepsilon(\lambda_{nm})$	Minimal condition
383.1865	-3.1	$C_{23}H_{26}O_5$	Ochrephilone	MS-Dial	0.8	Level 2	[7]	22000(415)	CZK
399.1805	-0.7	$C_{23}H_{26}O_6$		In-house	0.9	Level 3	[1]		CZK
417.1471	-1.9	$C_{23}H_{25}ClO_5$	Isochromophilone I	GNPS	0.71	Level 2	[8]	15700(412)	CZK

Level 0 corresponds annotations from isolated pure compounds; level 1 to annotations by comparison with standards; level 2 to putative annotations (e.g., MS/MS library comparison or tentative structure); level 3 to a chemical class assignment

Figure S2: Spectral comparison with databases of cluster B annotated molecules with their theoretical protonated *m/z*.

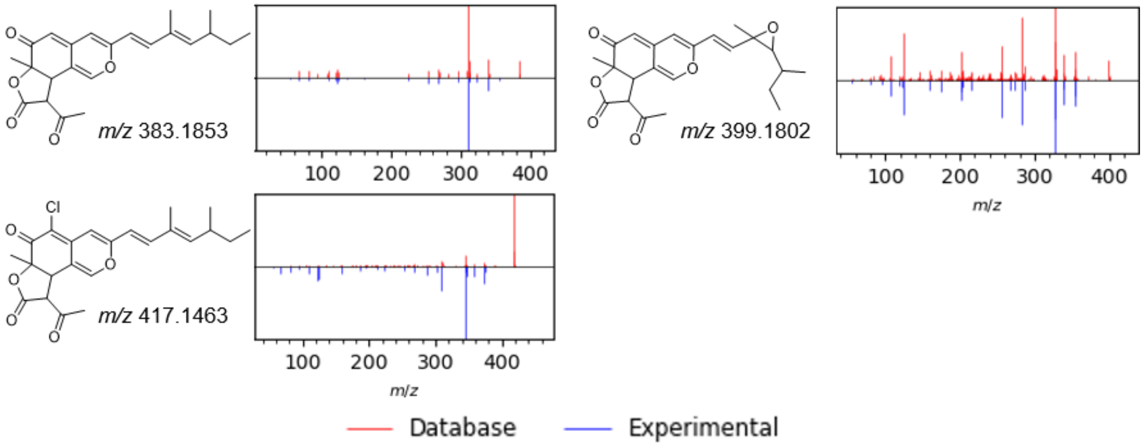

Table S3: Cluster C features annotated by MS/MS database query. In-house database was built upon annotations from Hebra et al.[1]

ClusterC									
$[M+H]^+$	Error (ppm)	Formula	Name	Database	Cosine score	Annotation	Ref	$\varepsilon(\lambda_{nm})$	Minimal condition
356.1859	-0.8	$C_{21}H_{25}NO_4$		In-house	0.94	Level 2	[1]		PD
362.1527	-2.6	$C_{20}H_{24}ClNO_3$		In-house	0.94	Level 2	[1]		YE
390.1469	-0.6	$C_{21}H_{24}ClNO_4$	Sclerotioramine	MONA	0.94	Level 1	[1,9]	20 000(331)	PD
404.1626	-0.7	$C_{22}H_{26}ClNO_4$		In-house	0.99	Level 2	[1]		PD
406.1420	-1.0	$C_{21}H_{24}ClNO_5$		In-house	0.95	Level 3	[1]		YE
418.1786	-1.5	$C_{23}H_{28}ClNO_4$		In-house	0.97	Level 3	[1]		YE
432.1946	-2.3	$C_{24}H_{30}ClNO_4$		In-house	0.93	Level 3	[1]		PD
434.1739	-2.4	$C_{23}H_{28}ClNO_5$		In-house	0.91	Level 3	[1]		PD

Level 0 corresponds annotations from isolated pure compounds; level 1 to annotations by comparison with standards; level 2 to putative annotations (e.g., MS/MS library comparison or tentative structure); level 3 to a chemical class assignment

Figure S3: Spectral comparison of cluster C molecules with databases with their theoretical protonated *m/z*.

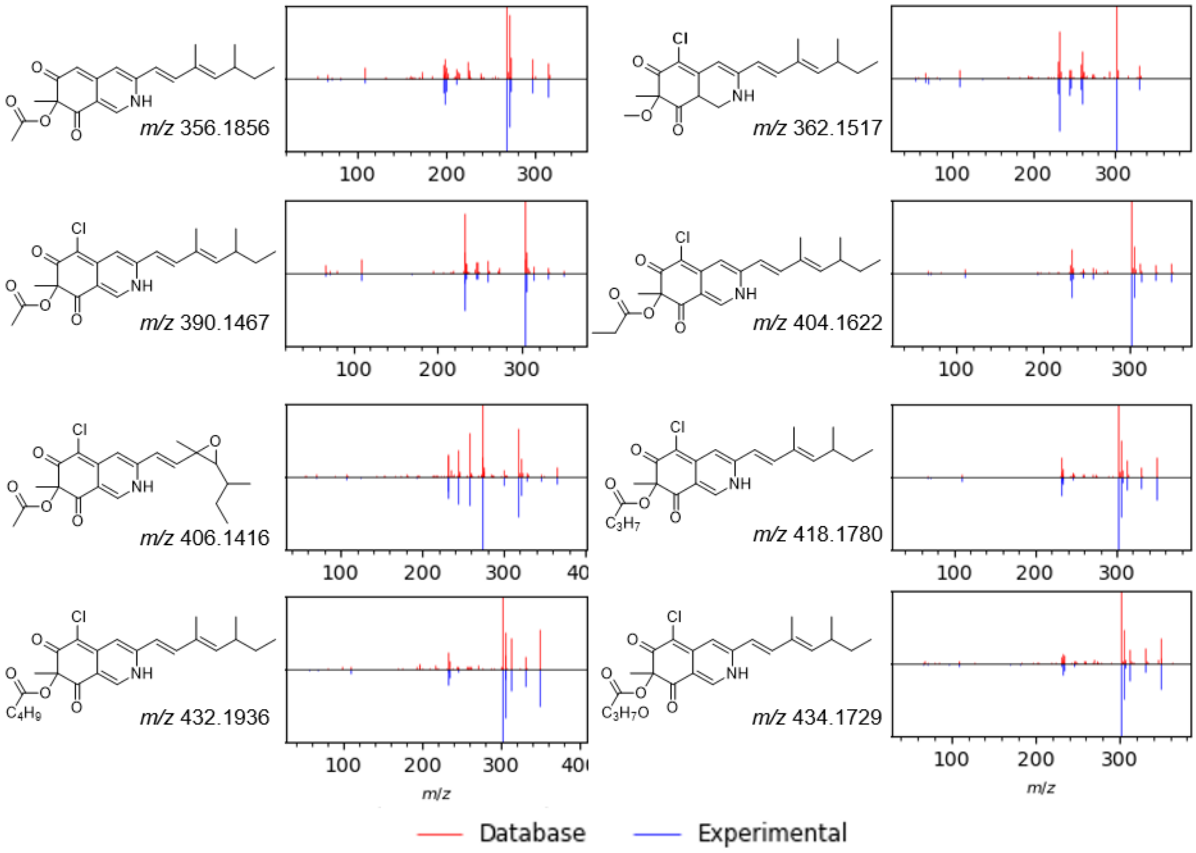

Table S4 : Cluster D features annotated by MS/MS database query. In-house database was built upon annotations from Hebra et al.[1]

ClusterD									
$[M+H]^+$	Error (ppm)	Formula	Name	Database	Cosine score	Annotation	Ref	$\varepsilon(\lambda_{nm})$	Minimal condition
400.2138	-4.9	$C_{23}H_{29}NO_5$		In-house	0.81	Level 2	[1]		PD
434.1732	-0.8	$C_{23}H_{28}ClNO_5$	Isochromophilone VI	MS-Dial	0.83	Level 1	[5]	23400(370)	CZK
448.1526	-1.0	$C_{23}H_{26}ClNO_6$		In-house	0.88	level 2	[1]		YE
448.1903	-4.0	$C_{24}H_{30}ClNO_5$		In-house	0.91	Level 2	[1]		YE
460.1530	-1.9	$C_{24}H_{26}ClNO_6$		In-house	0.87	Level 3	[1]		PD
476.1840	-1.2	$C_{25}H_{30}ClNO_6$	Isochromophilone IX	MONA	0.88	Level 2	[10]	No data	YE
478.1617	2.1	$C_{24}H_{28}ClNO_7$		In-house	0.9	Level 3	[1]		YE
490.1997	-1.2	$C_{26}H_{32}ClNO_6$		In-house	0.91	Level 3	[1]		YE
505.1737	-0.2	$C_{25}H_{29}ClN_2O_7$		In-house	0.93	Level 3	[1]		PD
519.1902	-1.8	$C_{26}H_{31}ClN_2O_7$		In-house	0.94	Level 3	[1]		YE
520.1735	-0.5	$C_{26}H_{30}ClNO_8$		In house	0.83	Level 3	[1]		PD

Level 0 corresponds annotations from isolated pure compounds; level 1 to annotations by comparison with standards; level 2 to putative annotations (e.g., MS/MS library comparison or tentative structure); level 3 to a chemical class assignment

Figure S4: Spectral comparison of cluster D molecules with databases and their theoretical protonated *m*/*z*.

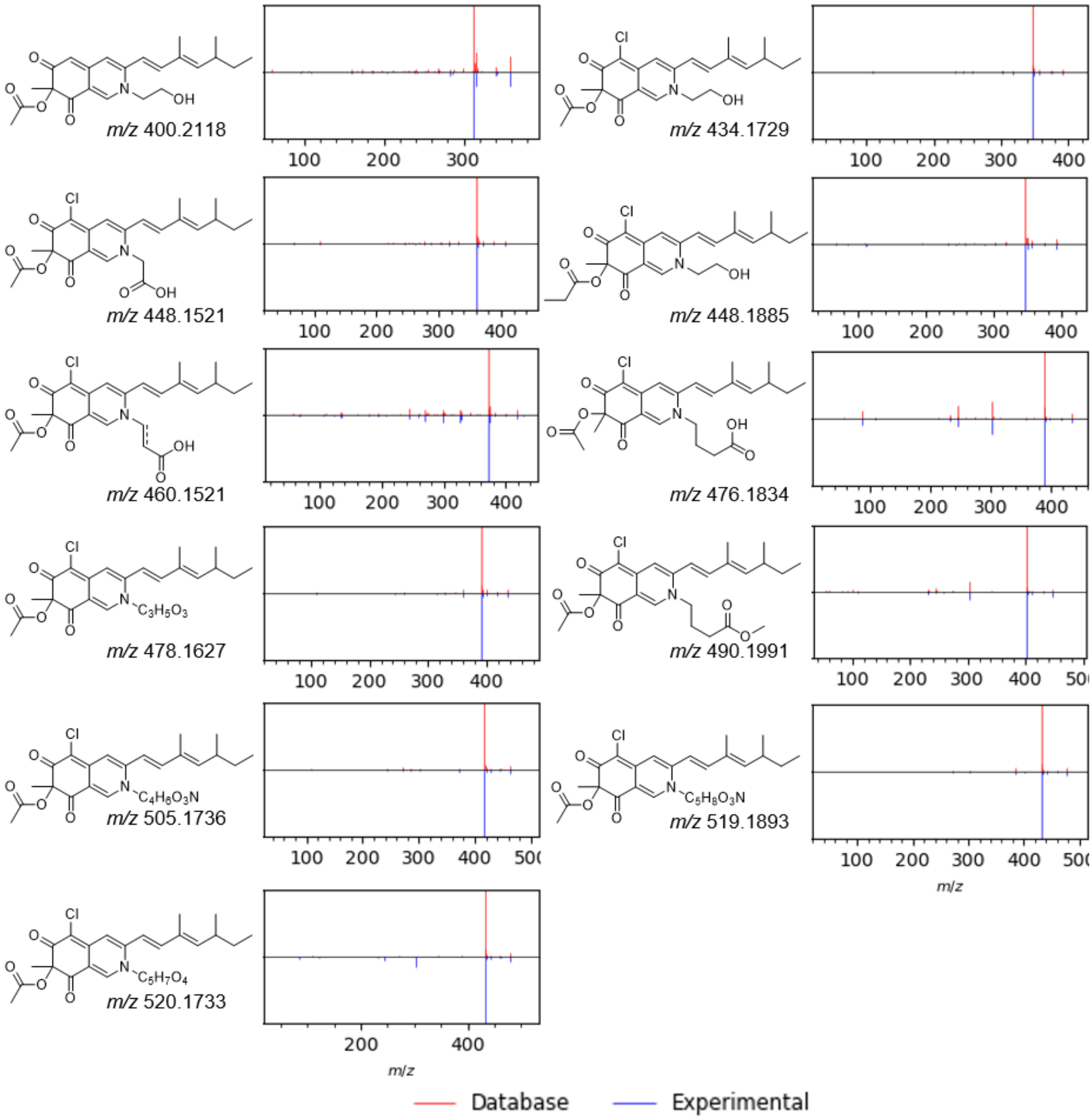

Table S5: Cluster E features annotated by MS/MS database query. In-house database was built upon annotations from Hebra et al.[1]

ClusterE									
$[M+H]^+$	Error (ppm)	Formula	Name	Database	Cosine score	Annotation	Ref	$\varepsilon(\lambda_{nm})$	Minimal condition
380.1860	-1.0	$C_{23}H_{25}NO_4$	Peniazaphilone E	In house	0.93	Level 1	[1]	9100(548)	PD
381.1705	-2.2	$C_{23}H_{24}O_5$		In-house	0.79	Level 2	[1]		PD
392.1618	1.3	$C_{21}H_{26}ClNO_4$	Deacetyl-isochromophilone VI	In-house	0.91	Level 1	[1]		YE
424.2119	-0.1	$C_{25}H_{29}NO_5$	Peniazaphilone A	In-house	0.93	Level 1	[1,11]	10900(541)	CZK

Level 0 corresponds annotations from isolated pure compounds; level 1 to annotations by comparison with standards; level 2 to putative annotations (e.g., MS/MS library comparison or tentative structure); level 3 to a chemical class assignment

Figure S5: Spectral comparison of cluster E molecules with databases and their theoretical protonated *m/z*.

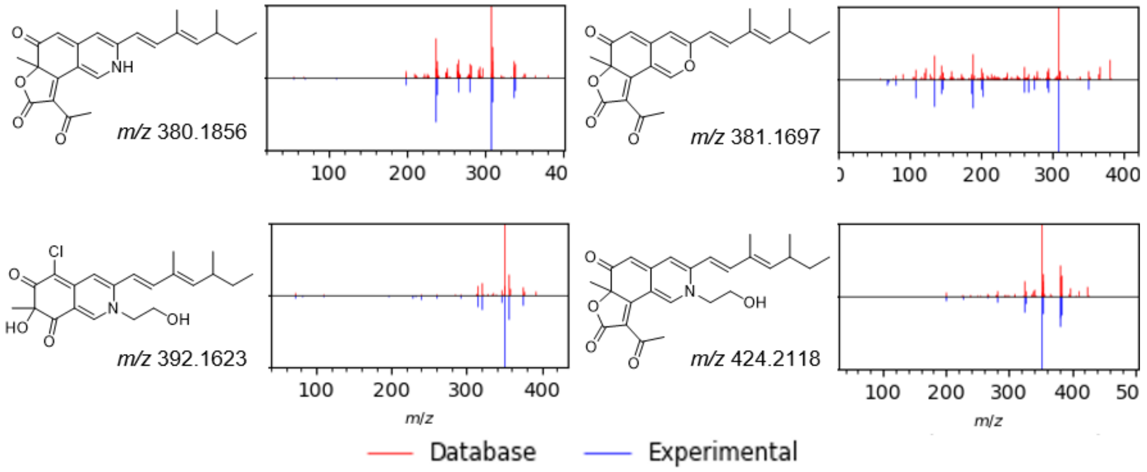

Table S6: Annotation of azaphilones from cluster D-I with proposed chain structure from molecular network data and KEGG compound database.[12]

Cluster D-I							
**ID**	**[M+H]⁺**	**Error (ppm)**	**Chain Formula**	**Kegg_ID**	**Chain**	**Acylation**	**Supplementary information**
**4**	434.1732	-0.8	C_2H_7NO	C00189	Ethanolamine	Acetyl	
**6**	400.2138	-4.9	C_2H_7NO	C00189	Ethanolamine	Acetyl	No Cl on azaphilone scaffold
**7**	404.1635	-3.0	CH_5N	C00218	Methylamine	Acetyl	
**8**	446.1734	-1.2	C_3H_7NO	C01888<br>C05665	Aminoacetone<br>3-Aminopropanal	Acetyl	
**9**	446.1746	-3.9	C_3H_7NO	C01888<br>C05665	Aminoacetone<br>3-Aminopropanal	Acetyl	
**10**	448.1526	-1.0	$C_2H_5NO_2$	C00037	Glycine	Acetyl	
**11**	448.1901	-3.5	C_2H_7NO	C00189	Ethanolamine	Propionyl	
**12**	460.1530	-1.9	$C_3H_5NO_2$	C02218<br>C20253	Dehydroalanine<br>Aminoacrylate	Acetyl	
**13**	460.1901	-3.4	C_3H_7NO	C01888<br>C05665	Aminoacetone<br>3-Aminopropanal	Propionyl	
**14**	460.1901	-3.4	C_4H_9NO	C00555	4-Aminobutanal	Acetyl	
**15**	460.2261	-2.6	$C_5H_{13}N$	C02640	Isoamylamine	Acetyl	
**16**	462.1690	-2.6	$C_3H_7NO_2$	C00041	Alanine	Acetyl	
**17**	474.2052	-2.2	$C_5H_{11}NO$	C01842<br>C12455	Pentanamide<br>5-Aminopentanal	Acetyl	
**18**	474.2054	-2.6	$C_5H_{11}NO$	C01842<br>C10974	Pentanamide<br>4-Methylaminobutanal	Acetyl	
**19**	476.1834	-0.1	$C_4H_9NO_2$		Methoxyacetamide	Acetyl	
**20**	476.1840	-1.2	$C_4H_9NO_2$	C00334	4-Aminobutanoate	Acetyl	
**21**	478.1617	2.1	$C_3H_7NO_3$	C00065	Serine	Acetyl	
**22**	490.1997	-1.2	$C_5H_{11}NO_2$	C00183	Valine	Acetyl	
**23**	501.2175	-4.9	$C_6H_{12}N_2O$	C19809	Piperidine-2-carboxamide	Acetyl	
**24**	504.2157	-1.9	$C_6H_{13}NO_2$	C00123<br>C00407	Leucine<br>Isoleucine	Acetyl	
**25**	505.1740	-0.8	$C_4H_8N_2O_3$	C00152	Asparagine	Acetyl	
**26**	518.2314	-2.0	$C_6H_{13}NO_2$	C00123<br>C00407	Leucine<br>Isoleucine	Propionyl	
**27**	519.1902	-1.8	$C_5H_{10}N_2O_3$	C00064	Glutamine	Acetyl	
**28**	519.2266	-1.9	$C_6H_{14}N_2O_2$	C00047	Lysine	Acetyl	
**29**	520.1735	-0.4	$C_5H_9NO_4$	C00025	Glutamate	Acetyl	
**30**	536.2054	-1.6	$C_6H_{13}NO_4$	C22301	Dihydroxyisoleucine	Acetyl	
**31**	538.2001	-1.9	$C_9H_{11}NO_2$	C00079	Phenylalanine	Acetyl	
**32**	547.2212	-1.2	$C_7H_{14}N_2O_3$	C00437	N-Acetylornithine	Acetyl	
**33**	550.2214	-2.2	$C_6H_{13}NO_4$	C22301	Dihydroxyisoleucine	Propionyl	
**34**	552.2173	4.6	$C_9H_{11}NO_2$	C00079	Phenylalanine	Propionyl	
**35**	554.1929	2.0	$C_9H_{11}NO_3$	C00082	Tyrosine	Acetyl	

Table S7: Annotation of azaphilones from cluster D-II with proposed chain structure from molecular network data and KEGG compound database.[12]

Cluster DII							
**ID**	**[M+H]$^+$**	**Error (ppm)**	**Chain Formula**	**Kegg_ID**	**Chain**	**Acylation**	**Supplementary information**
**36**	460.2493	-2.3	$C_8H_{11}N$	C02455	Phenylethylamine	Acetyl	No Cl on azaphilone scaffold
**37**	476.2427	1.0	$C_8H_{11}NO$	C00483<br>C01183	Tyramine<br>Phenylethanolamine	Acetyl	No Cl on azaphilone scaffold
**38**	484.2012	2.6	$C_5H_9N_3$	C00388	Histamine	Acetyl	
**39**	494.2105	-2.5	$C_8H_{11}N$	C02455	Phenylethylamine	Acetyl	
**40**	503.2301	1.3	$C_6H_{14}N_2O$	C02714	N-Acetylputrescine	Acetyl	
**41**	508.2254	-1.0	$C_8H_{11}N$	C02455	Phenylethylamine	Propionyl	
**42**	510.2046	-0.8	$C_8H_{11}NO$	C00483<br>C01183	Tyramine<br>Phenylethanolamine	Acetyl	
**43**	510.2055	-2.6	$C_8H_{11}NO$	C00483<br>C01183	Tyramine<br>Phenylethanolamine	Acetyl	
**44**	522.1721	-1.8	$C_5H_{11}NO_2S$	C00073	Methionine	Acetyl	
**45**	522.2418	-2.4	$C_8H_{11}N$	C02455	Phenylethylamine	Butyl	
**46**	524.2208	-1.9	$C_8H_{11}NO$	C00483<br>C01183	Tyramine<br>Phenylethanolamine	Propionyl	
**47**	526.1997	-1.2	$C_8H_{11}NO_2$	C03758<br>C04227	Dopamine<br>Octopamine	Acetyl	
**48**	540.2140	1.4	$C_8H_{11}NO_2$	C03758<br>C04227	Dopamine<br>Octopamine	Propionyl	

Table S8: Annotation of azaphilones from cluster D-III with proposed chain structure from molecular network data and KEGG compound database.[12]

Cluster DIII							
**ID**	**[M+H]$^+$**	**Error (ppm)**	**Chain Formula**	**Kegg_ID**	**Chain**	**Acylation**	**Supplementary information**
**49**	541.2108	-1.5	$C_8H_{12}N_2O_2$	C00534	Pyridoxamine	Acetyl	
**50**	577.2112	-2.1	$C_{11}H_{12}N_2O_2$	C00078	Tryptophane	Acetyl	

Figure S6: Subdivision of Cluster D based on annotated features

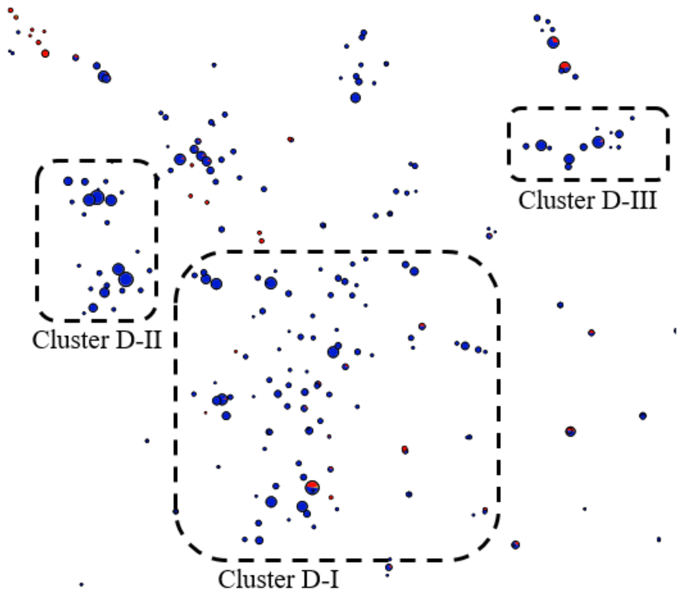

Table S9: Annotation of azaphilones from cluster E with proposed chain structure from molecular network data and KEGG compound database.[12]

Cluster E						
**ID**	**$[M+H]^+$**	**Error (ppm)**	**Chain Formula**	**Kegg_ID**	**Chain**	**Supplementary information**
**5**	380.186	-1.0	NH_3	C00014	Ammonium	
**51**	396.1809	-0.9	NH_3	C00014	Ammonium	Epoxy-azaphilone
**52**	424.2119	-0.1	C_2H_7NO	C00189	Ethanolamine	
**53**	436.212	-0.4	C_3H_7NO	C01888<br>C05665	Aminoacetone<br>3-Aminopropanal	
**54**	450.2646	-1.6	$C_5H_{13}N$	C02640	Isoamylamine	
**55**	484.2491	-1.8	$C_8H_{11}N$	C02455	Phenylethylamine	
**56**	494.2551	-2.8	$C_6H_{13}NO_2$	C00123<br>C00407	Leucine/Isoleucine	
**57**	500.2439	-1.5	$C_8H_{11}N$	C02455	Tyramine<br>Phenylethanolamine	Epoxy-azaphilone
**58**	500.2445	-2.7	$C_8H_{11}NO$	C00483<br>C01183	Tyramine<br>Phenylethanolamine	
**59**	516.2394	-2.6	$C_8H_{11}NO$	C00483<br>C01183	Tyramine<br>Phenylethanolamine	Epoxy-azaphilone
**60**	528.2386	-1.0	$C_9H_{11}NO_2$	C00079	Phenylalanine	
**61**	534.2005	7.0	$C_8H_{11}NO$	C00483<br>C01183	Tyramine<br>Phenylethanolamine	Chlorination
**62**	567.2502	-2.2	$C_{11}H_{12}N_2O_2$	C00078	Tryptophane	

Figure S7: Annotation of cluster E with hypothetical chain structures integrated to the azaphilone scaffold, proposed from molecular network data and KEGG compound database.[12] epoxy means The 3,5-dimethyl-1,3-heptadienyl unit bear an epoxide on position 3. Cl means azaphilone is chlorinated

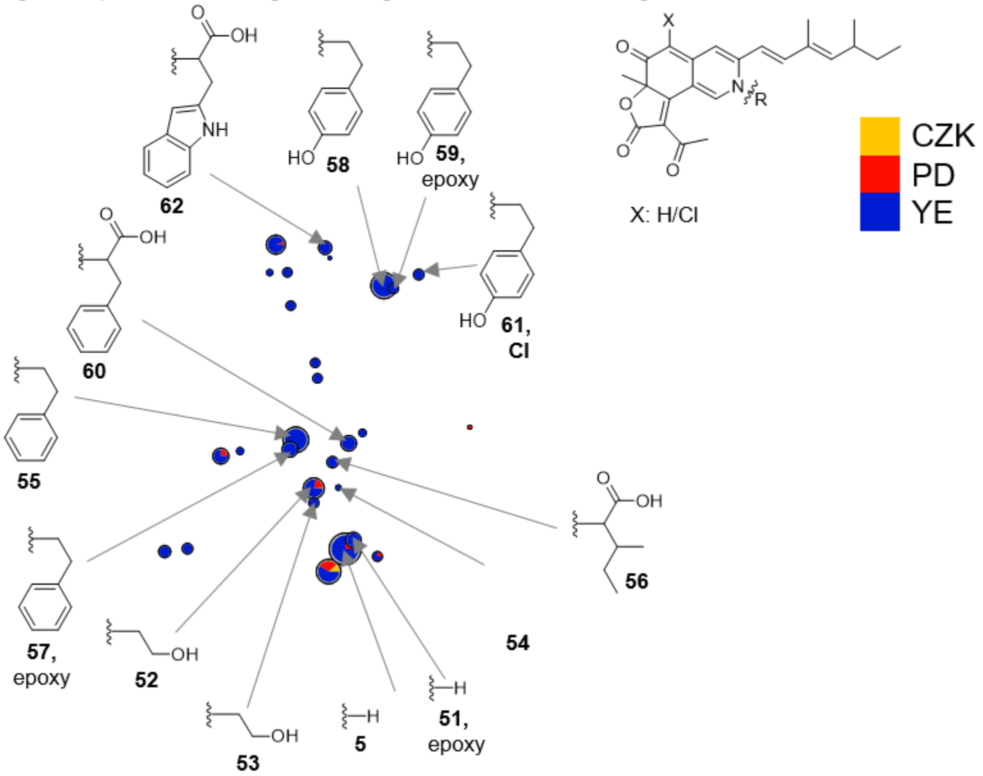

Figure S8: HRMS spectrum for compound **24’**

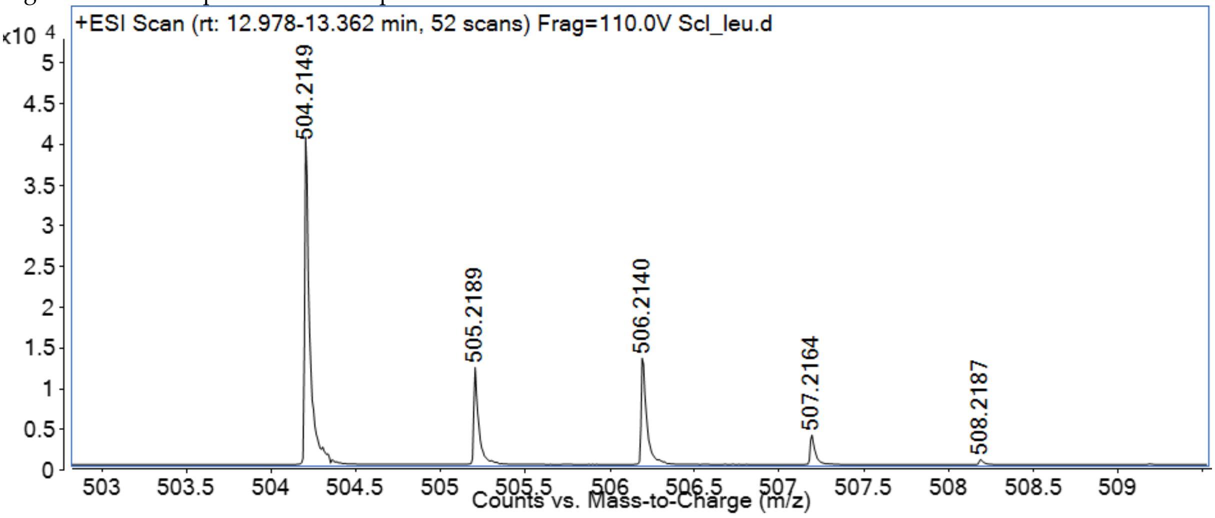

Figure S9: ^{1}H NMR spectrum (500 MHz, CD_3CN) of compound **24′**

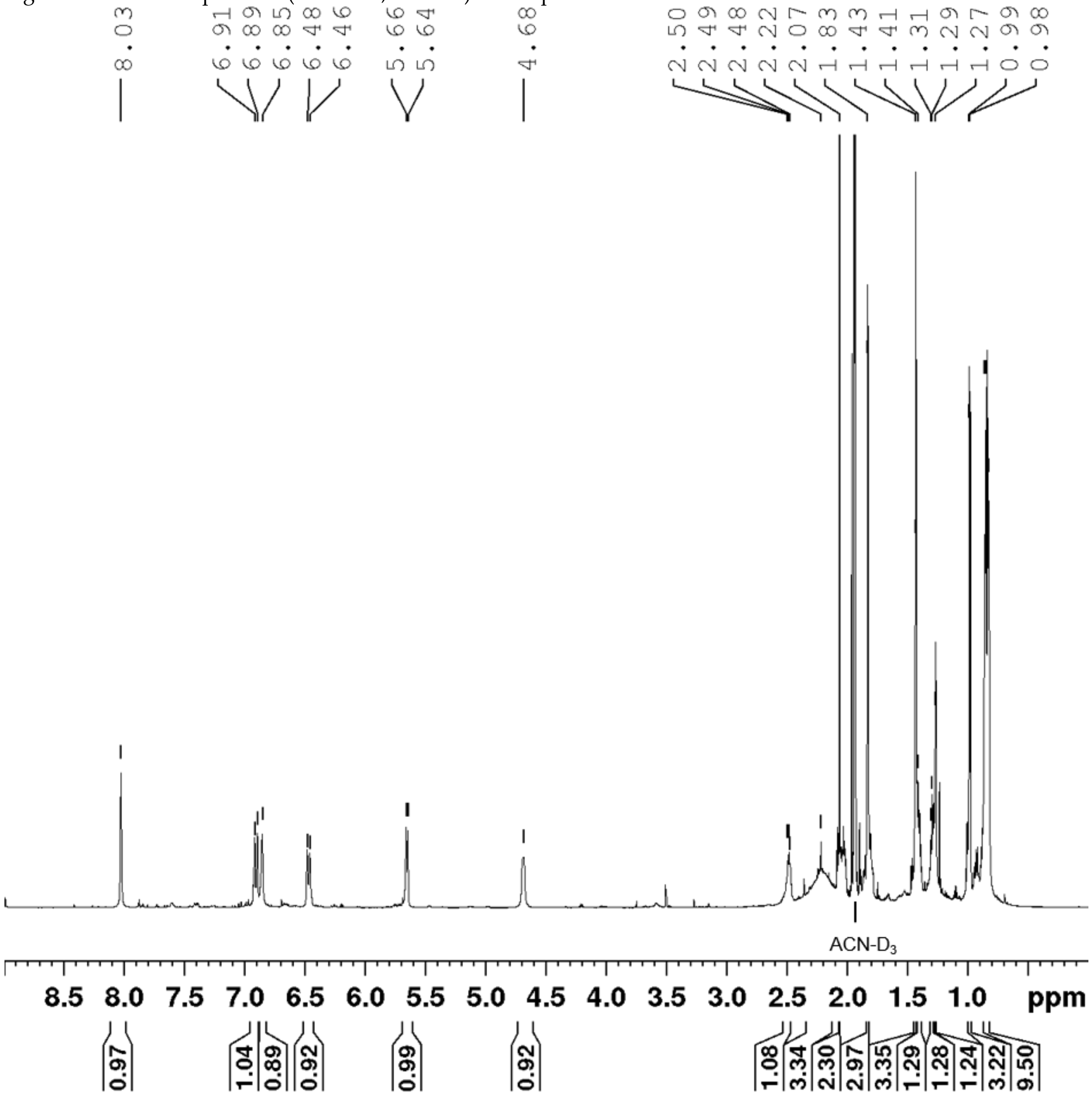

Figure S10: ^{13}C NMR spectrum (700 MHz, CD_3CN) of compound **24'**

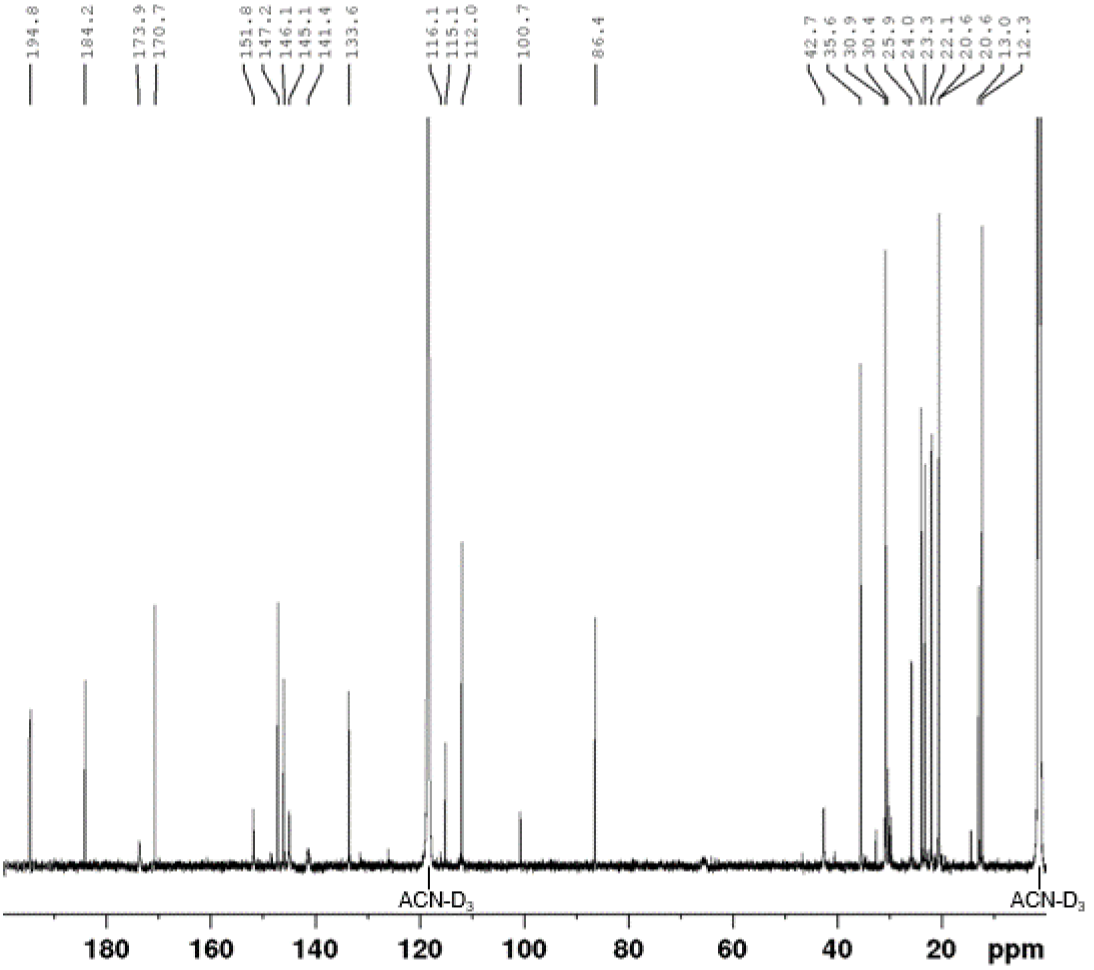

Figure S 11: HRMS spectrum for compound **39’**

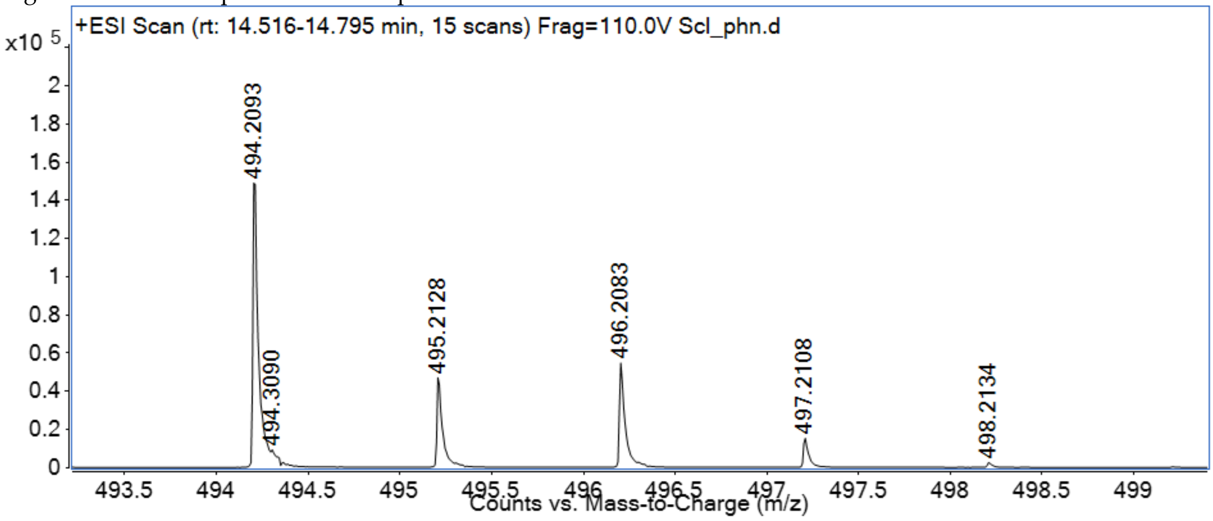

Figure S12: ^{1}H NMR spectrum (500 MHz, $CDCl_3$) of compound **39′**

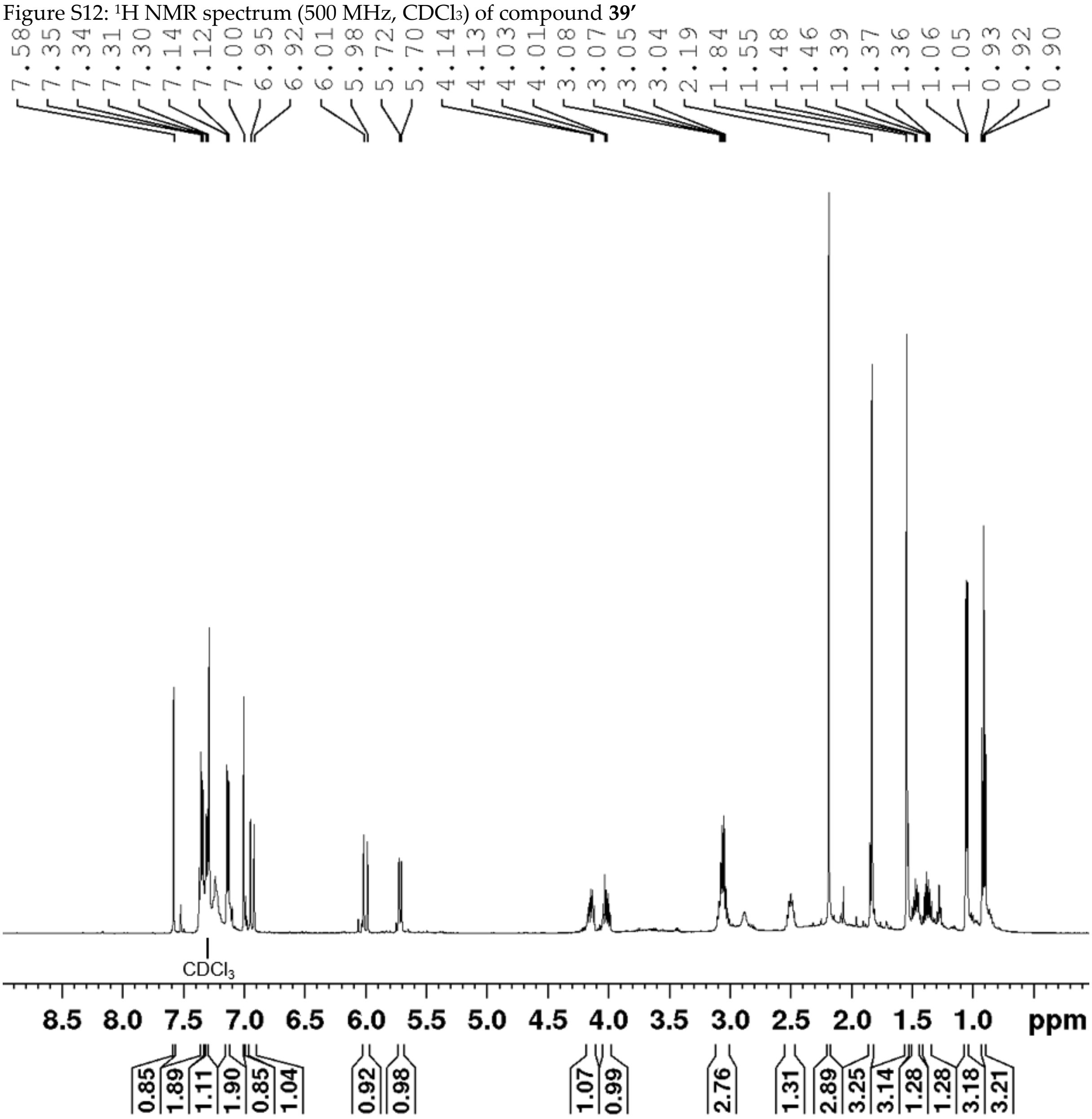

Figure S13: ^{13}C NMR spectrum (500 MHz, $CDCl_3$) of compound **39′**

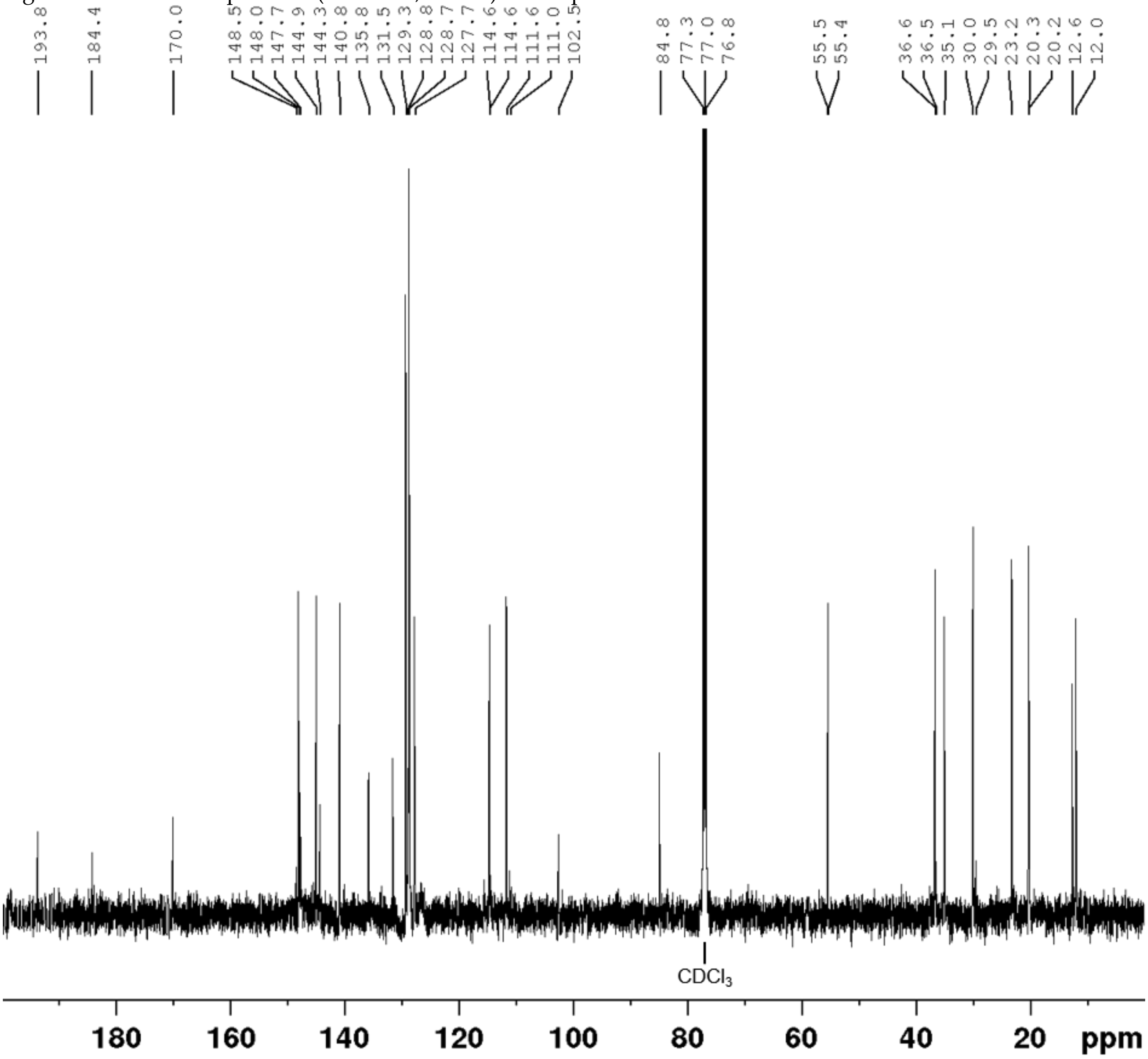

Figure S14: HRMS spectrum for compound **50′**

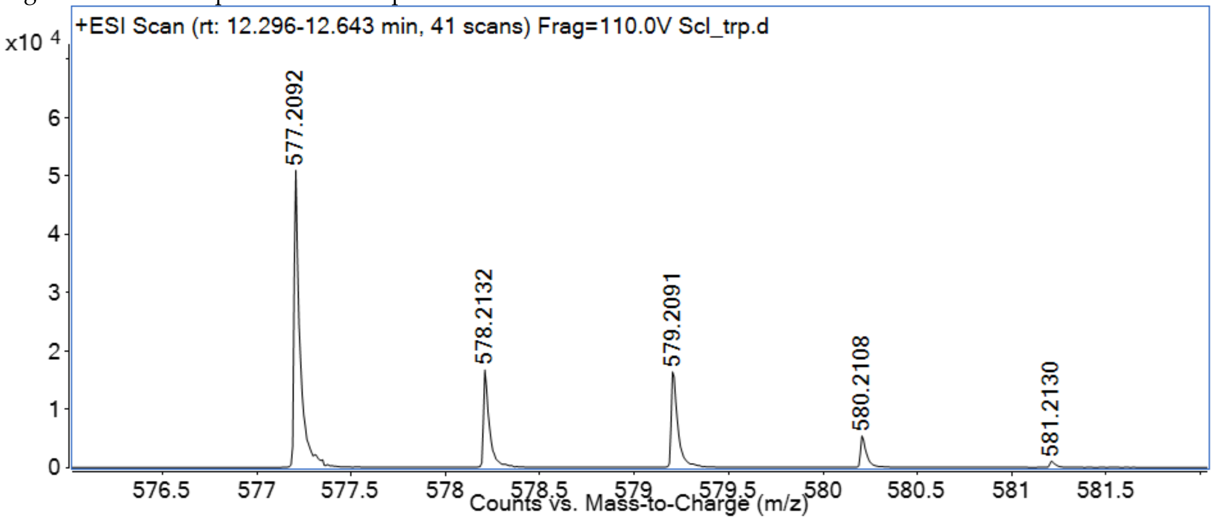

Figure S15: ^{1}H NMR spectrum (500 MHz, MeOD) of compound **50′**

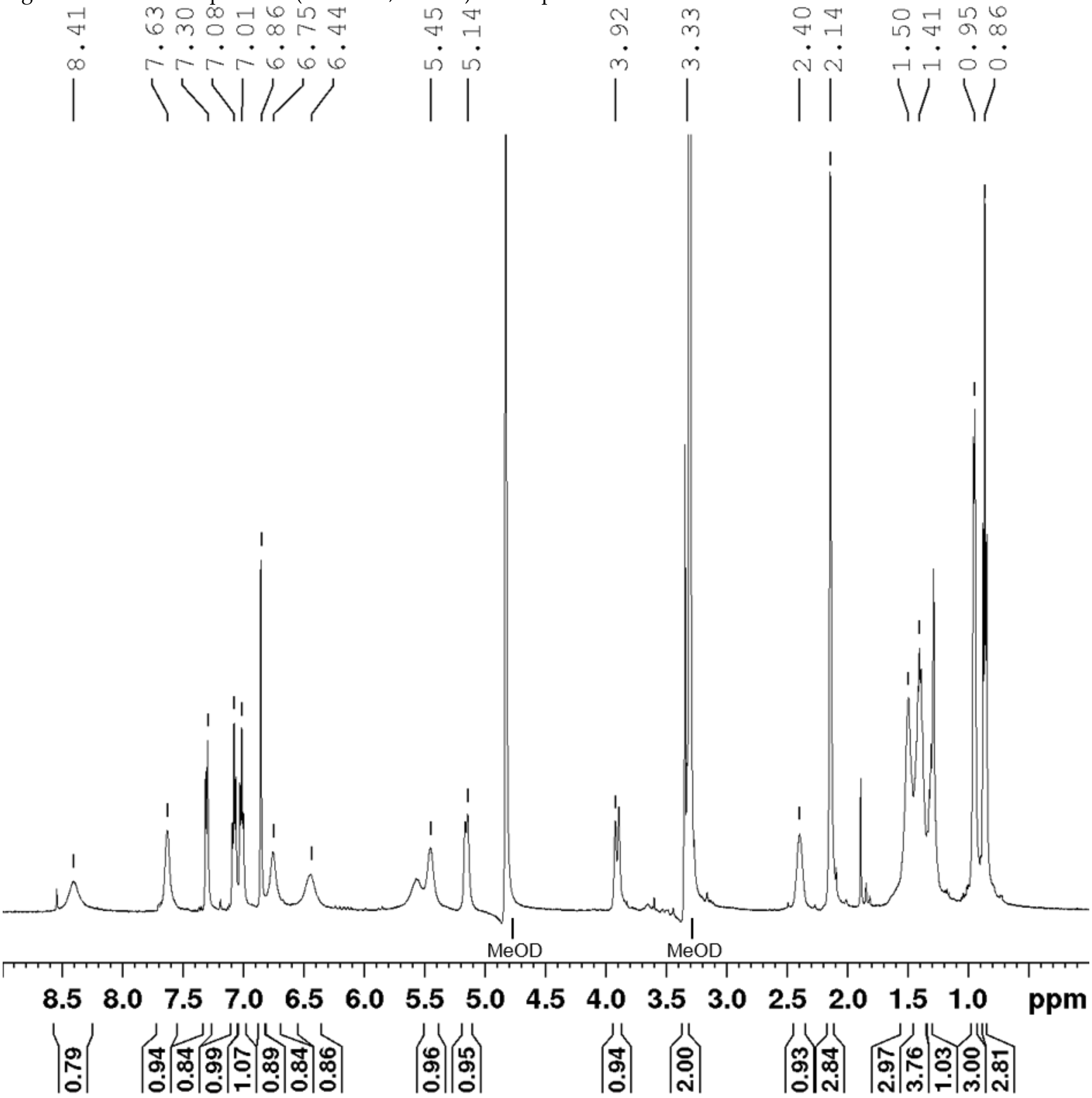

Figure S16: ^{13}C NMR spectrum (500, MHz, MeOD) of compound **50′**

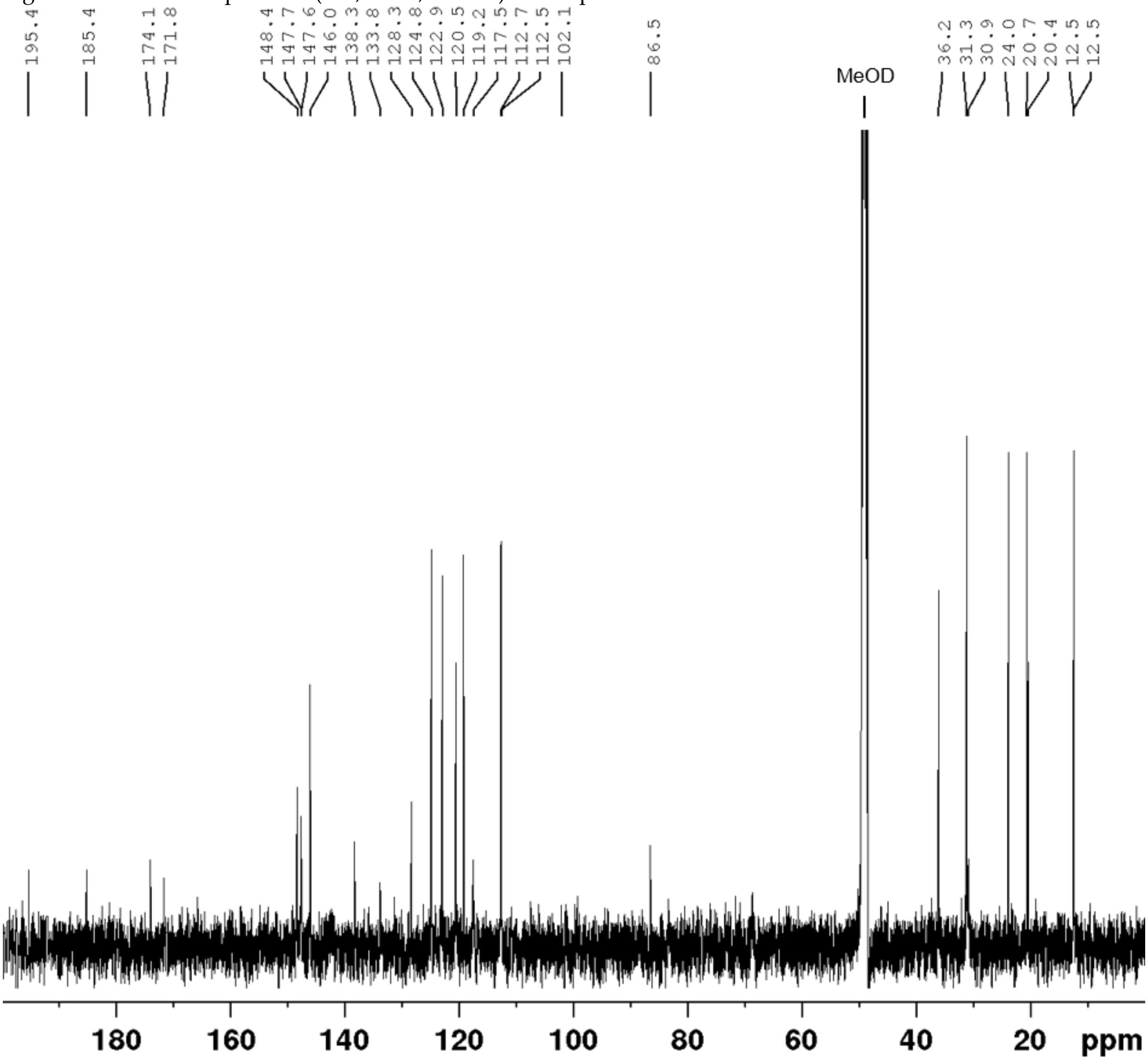

Figure S17: Comparison of fragmentation spectra and retention time from hemisynthetized standards with metabolites **24**, **39** and **50** produced by *P. sclerotiorum* SNB-CN111.

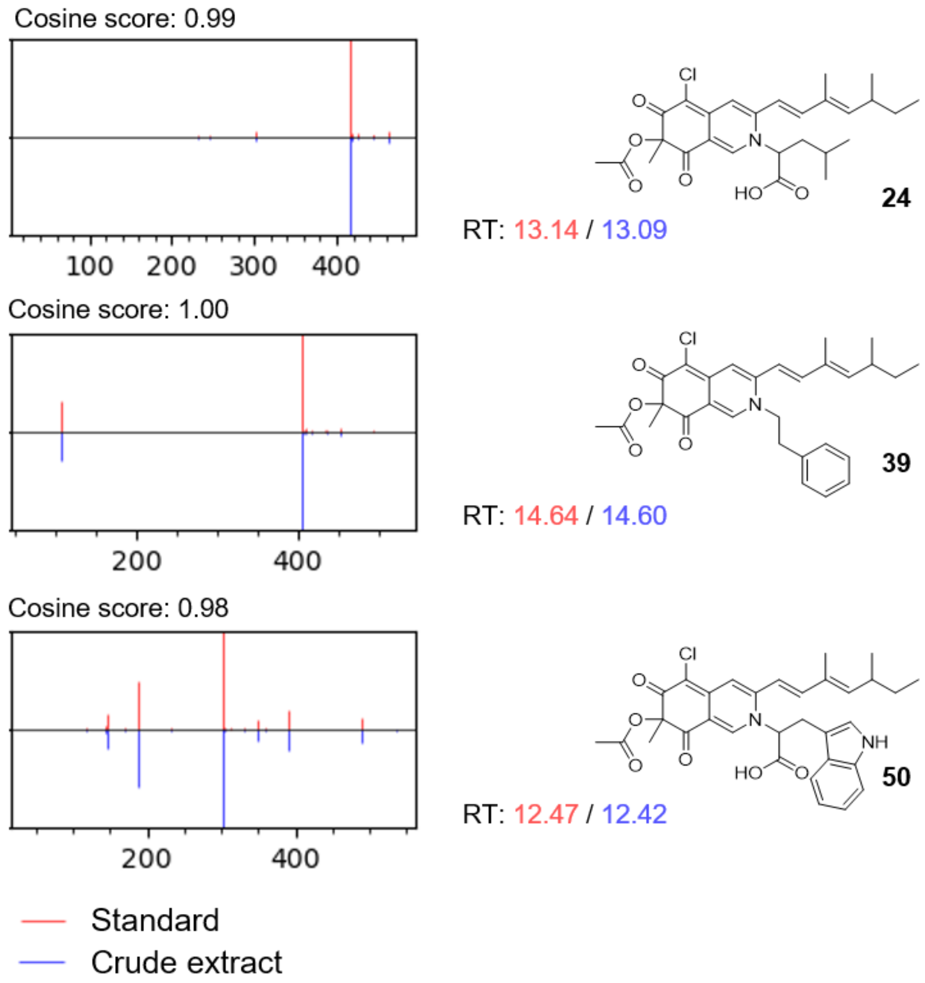

Table S10: Absorbance at 500, 460 and 400 nm of *P. sclerotiorum* SNB-CN111 crude extracts from fermentation on solid CZK, PD and YE. Values are given in $AU.g^{-1}$ of culture medium.

$AU_{nm}.g^{-1}$	CZK	PD	YE
OD_{500}	0.4	6.0	59.6
OD_{460}	1.0	10.4	64.9
OD_{400}	4.9	47.5	213.2

Table S11: Absorbance at 500, 460 and 400 nm of *P. sclerotiorum* SNB-CN111 crude extracts from fermentation on solid CZK supplemented with ammonium acetate or ethanolamine at 0, 0.1, 0.5 and 2 $g.L^{-1}$. Values are given in $AU.g^{-1}$ of culture medium.

	CZK + ammonium acetate ($g.L^{-1}$)				CZK + ethanolamine ($g.L^{-1}$)			
$AU_{nm}.g^{-1}$	0	0.1	0.5	2	0	0.1	0.5	2
OD_{500}	0.4	0.5	0.4	0.2	0.4	0.5	0.5	0.4
OD_{460}	1.0	1.1	0.8	0.6	1.0	1.1	1.1	0.7
OD_{400}	4.9	4.2	2.8	2.7	4.9	4.0	4.4	2.4

Figure S18: Extracted chromatogram for molecules **1** ([M+H]+, *m/z* 391.1307), **3** ([M+H]+, *m/z* 390.1467) and **4** ([M+H]+, *m/z* 434.1729) as a function of the concentration of ammonium acetate or ethanolamine added to solid CZK medium.

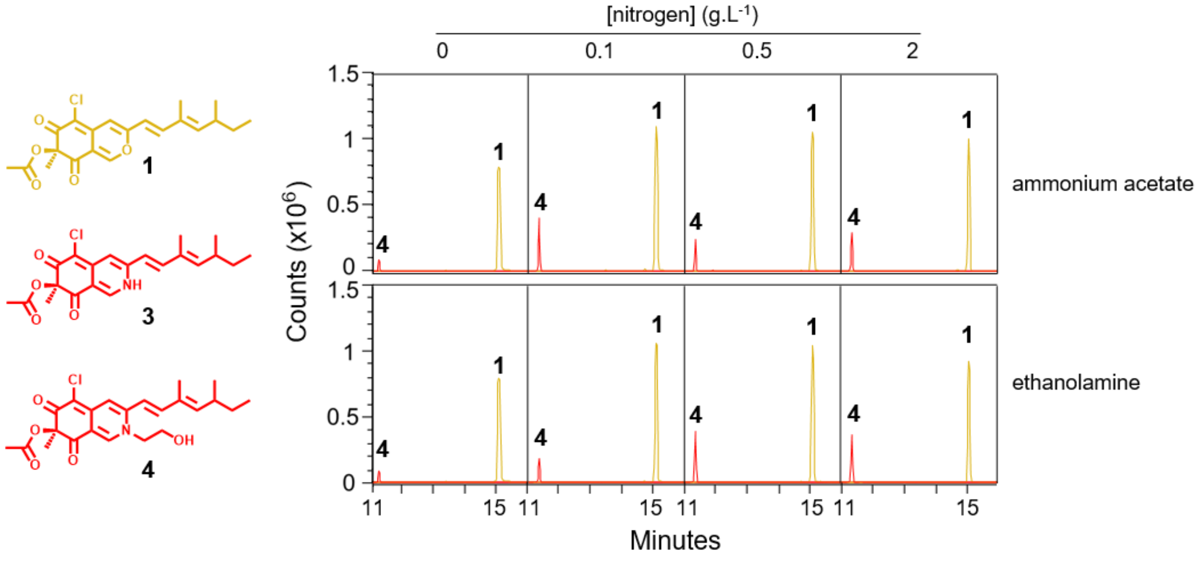

Table S12: Absorbance at 500, 460 and 400 nm of *P. sclerotiorum* SNB-CN111 crude extracts from two step fermentation process on porous cellophane membrane on solid CZK supplemented with ammonium acetate or ethanolamine at 0, 0.1, 0.5 and 2 g.L^{-1}. Values are given in AU.g^{-1} of culture medium.

	Two step fermentation CZK + ammonium acetate (g.L^{-1})				Two step fermentation CZK + ethanolamine (g.L^{-1})			
$AU_{nm}.g^{-1}$	0	0.1	0.5	2	0	0.1	0.5	2
OD_{500}	0.4	0.9	1.6	4.5	0.4	1.8	2.2	4.6
OD_{460}	1.0	2.1	3.2	10.3	1.0	4.2	5.1	8.9
OD_{400}	4.9	10.3	11.8	37.1	4.9	16.1	21.2	40.0

Table S13: Absorbance at 500, 460, and 400 nm of *P. sclerotiorum* SNB-CN111 crude extracts from two step fermentation process on porous cellophane membrane on solid CZK supplemented with propargilamine at 0, 0.1, 0.5 and 2 g.L^{-1}. Values are given in AU.g^{-1} of culture medium.

	Two step fermentation CZK + propargilamine (g.L^{-1})			
$AU_{nm}.g^{-1}$	0	0.1	0.5	2
OD_{500}	0.4	22.5	68.0	49.9
OD_{460}	1.0	38.3	91.7	68.2
OD_{400}	4.9	155.1	335.4	258.6

Figure S19: Metabolic profile of from *P. sclerotiorum* SNB-CN111 fermentation on solid CZK crude extract and two step fermentation process on porous cellophane membrane on solid CZK supplemented with propargilamine 0.5 g.L^{-1}.

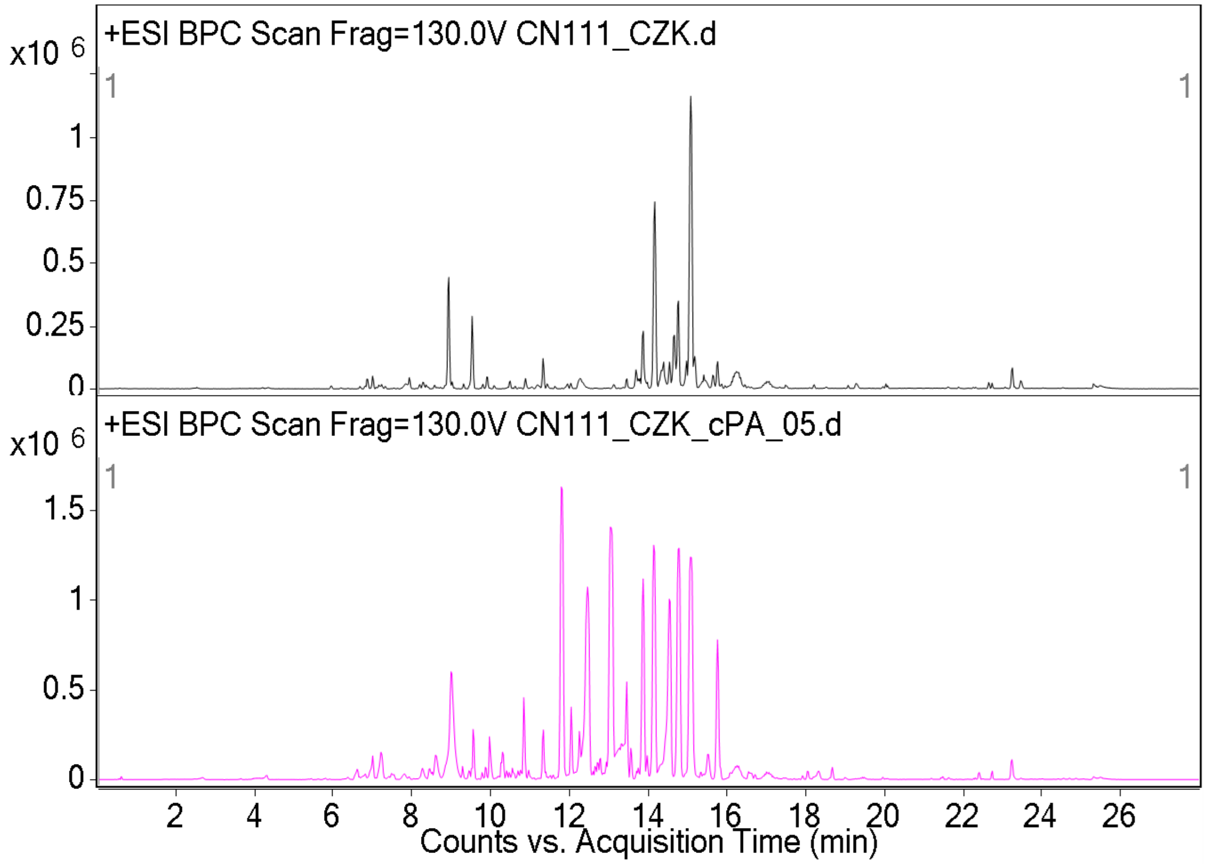

Figure S20: Base pic chromatogram of fraction F1 from flash chromatography of pulled two step fermentation process on porous cellophane membrane on solid CZK supplemented with propargilamine crude extracts.

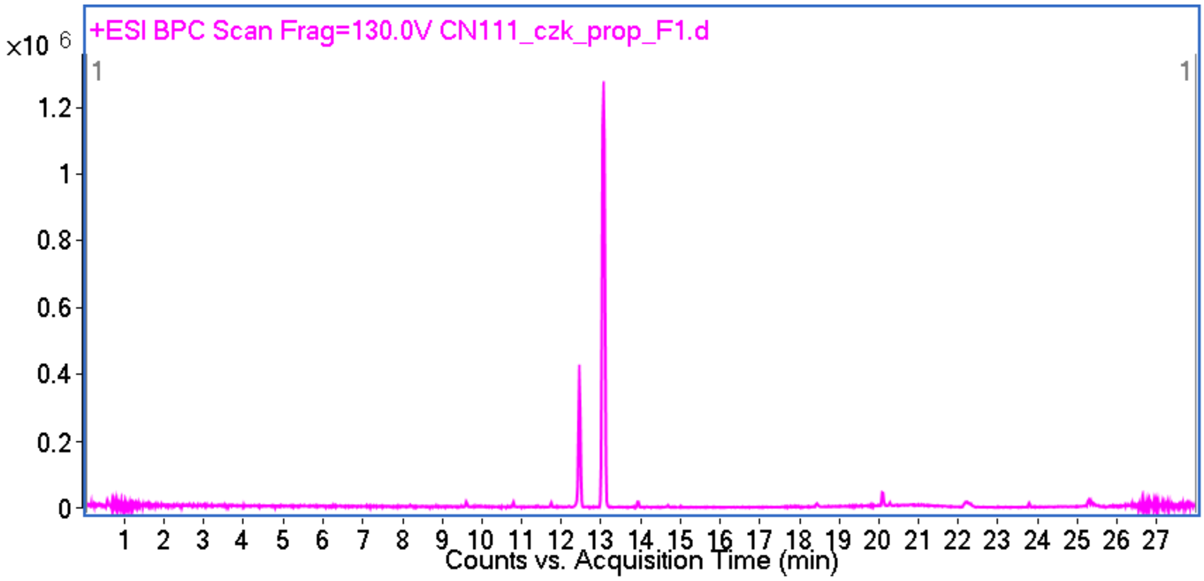

Figure S21 HRMS of compound **63**.

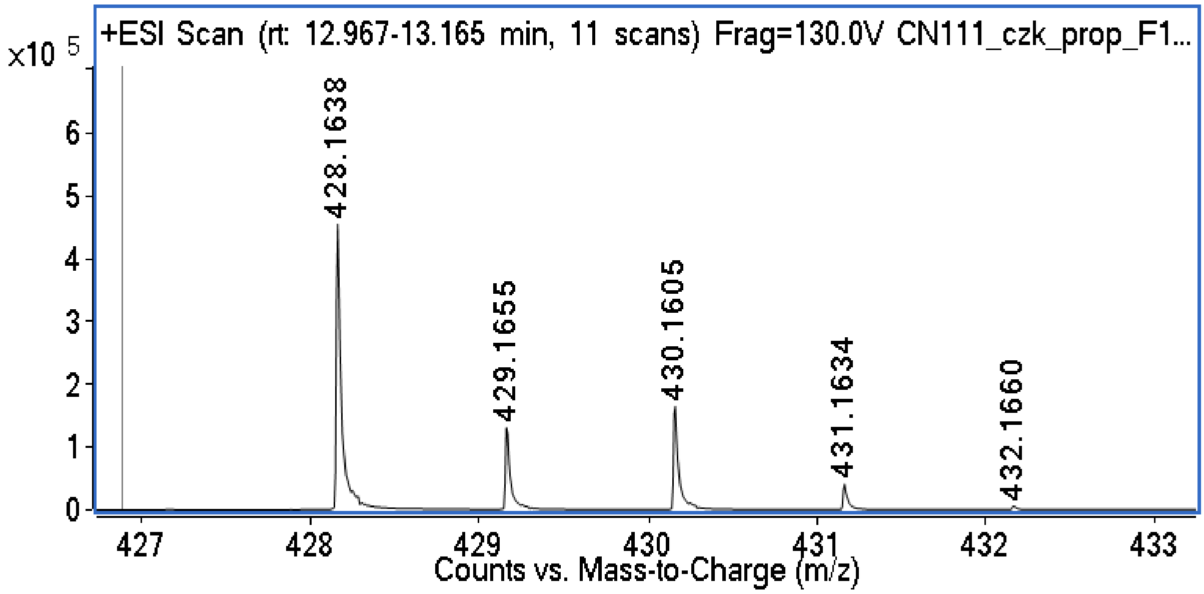

Figure S22: ^{1}H NMR spectrum (500 MHz, $CDCl_3$) of compound **63**.

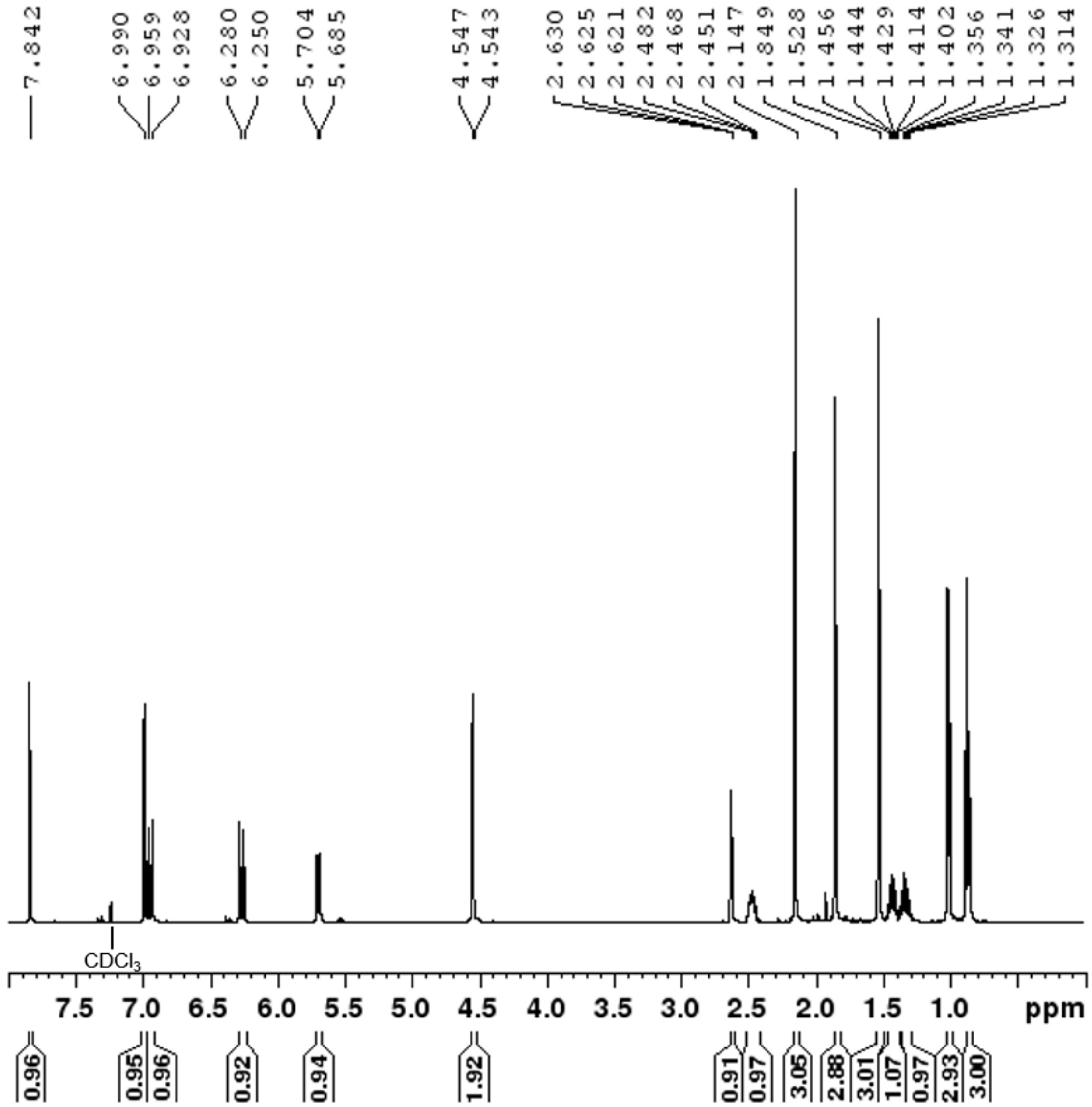

Figure S23: ^{13}C NMR spectrum (500 MHz, $CDCl_3$) of compound **63**.

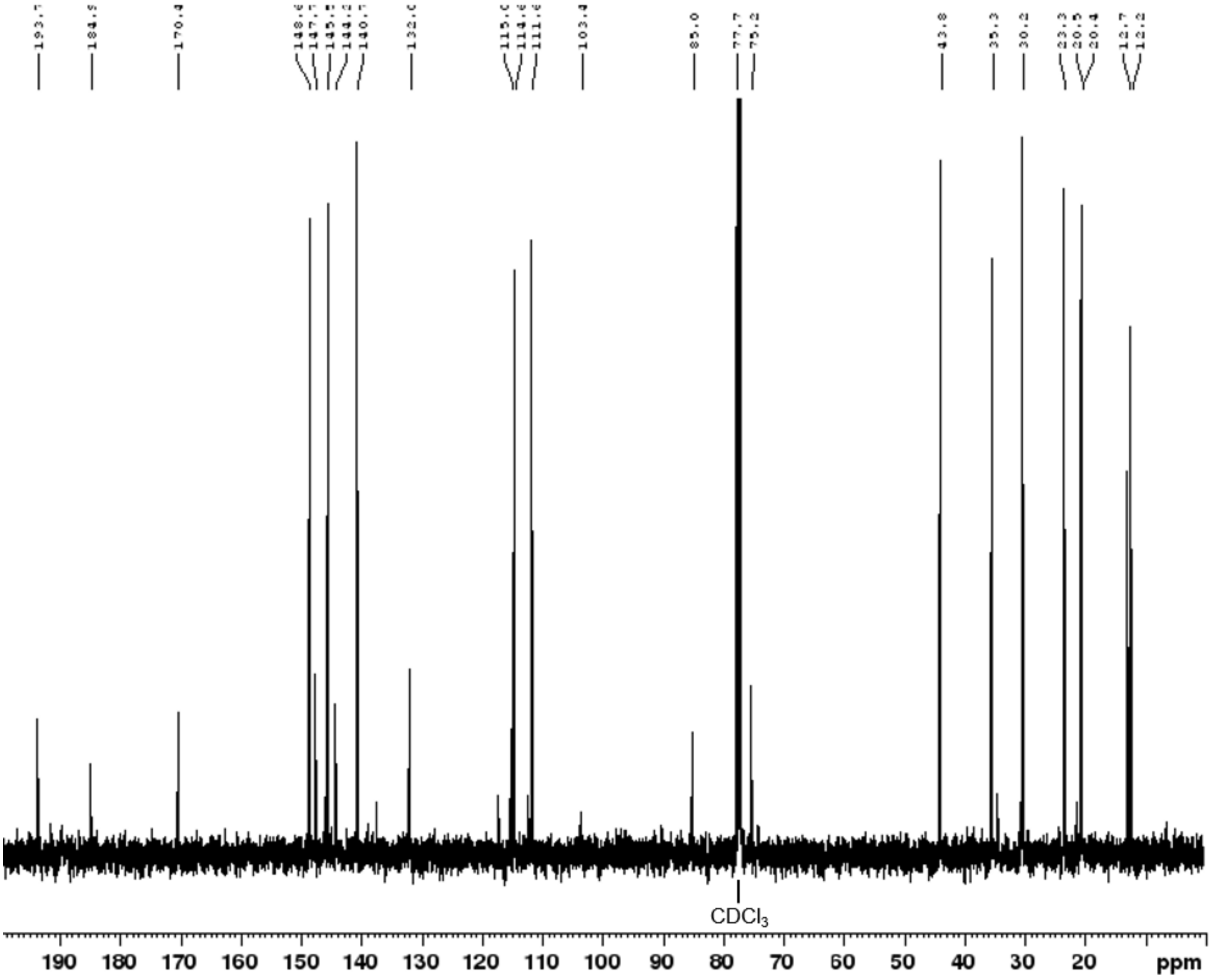

Figure S 24: COSY NMR spectrum (500 MHz, $CDCl_3$) of compound **63**.

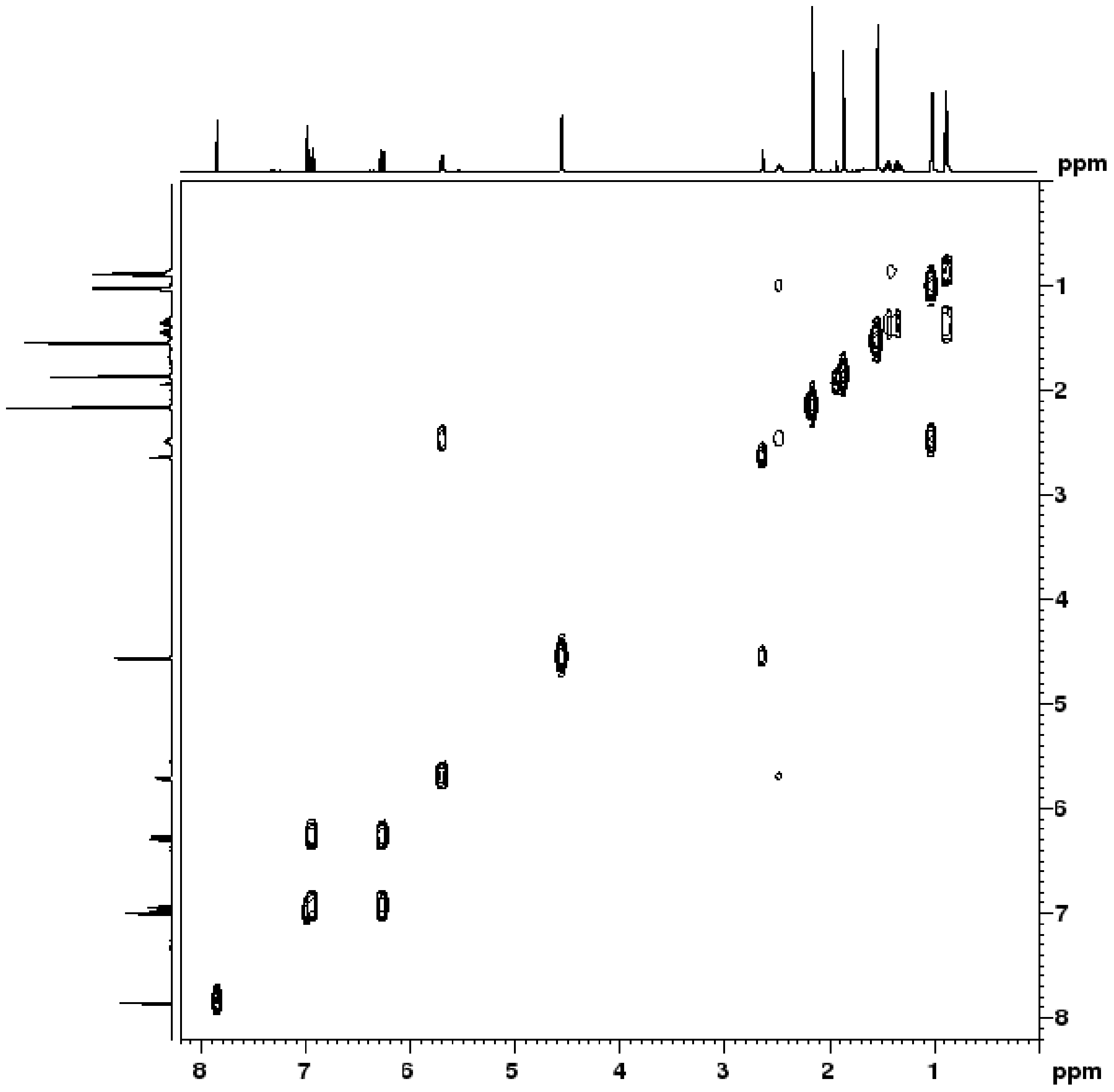

Figure S 25: HSQC NMR spectrum (500 MHz, $CDCl_3$) of compound **63**.

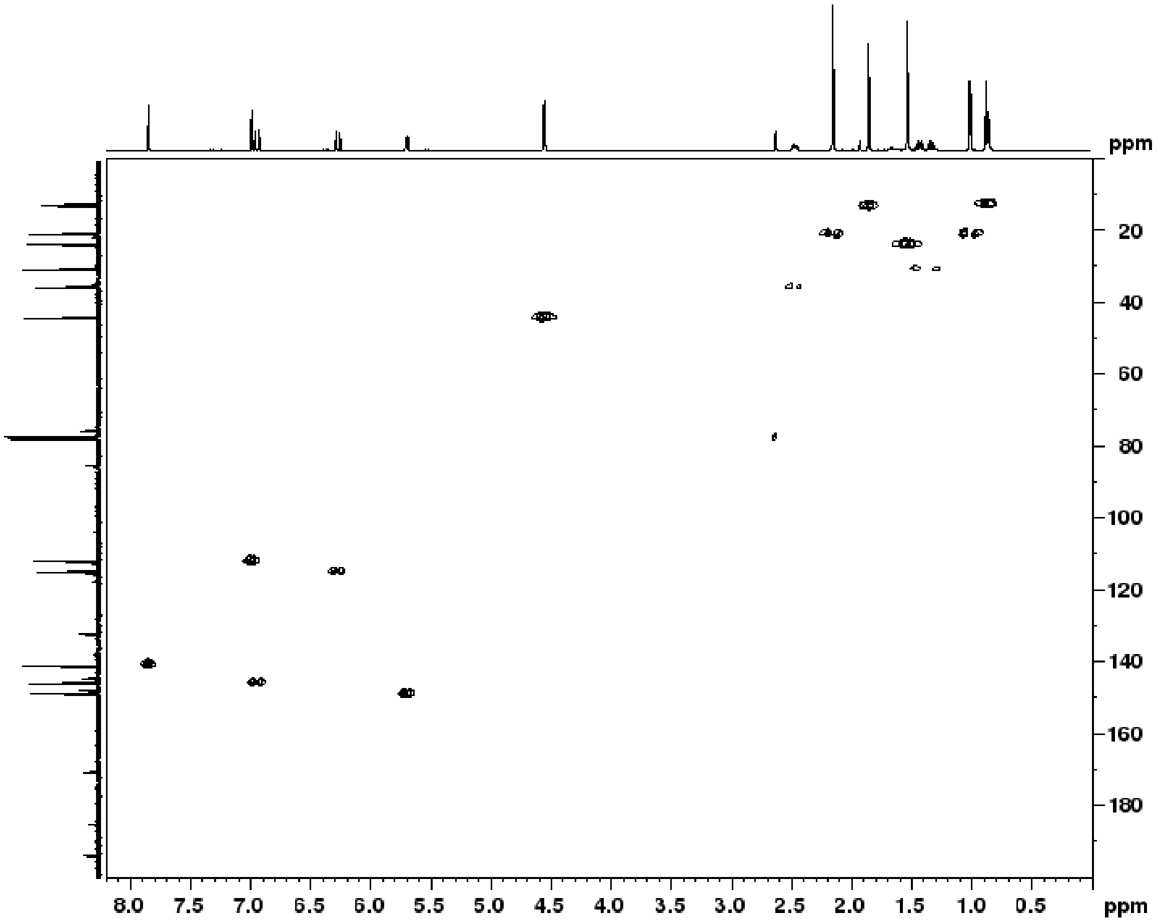

Figure S 26 HMBC NMR spectrum (500, MHz, $CDCl_3$) of compound **63**.

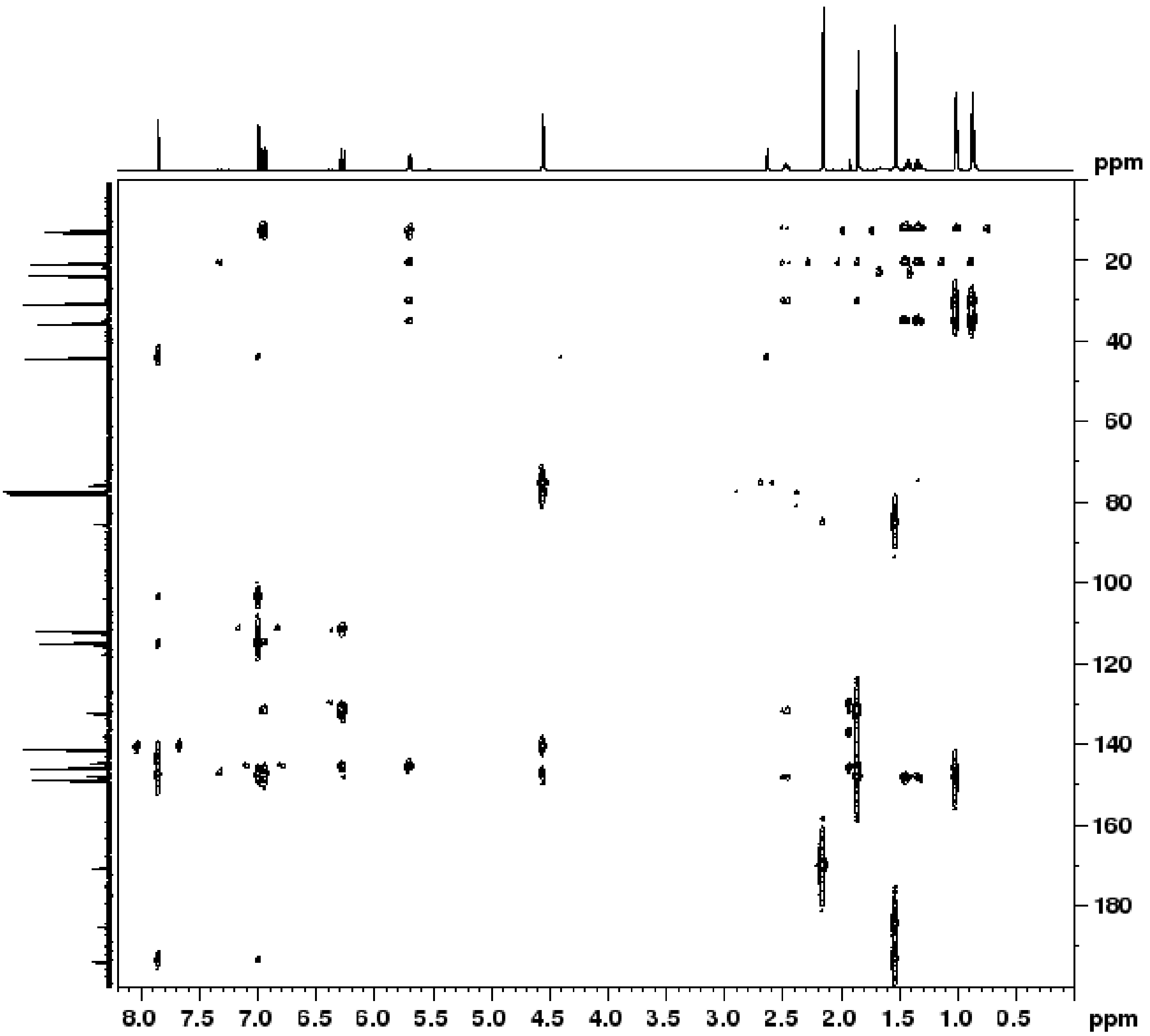

Figure S27: Key ^1H–^1H COSY (bold bonds) and ^1H–^{13}C (plain arrow) correlations of compound **63.**

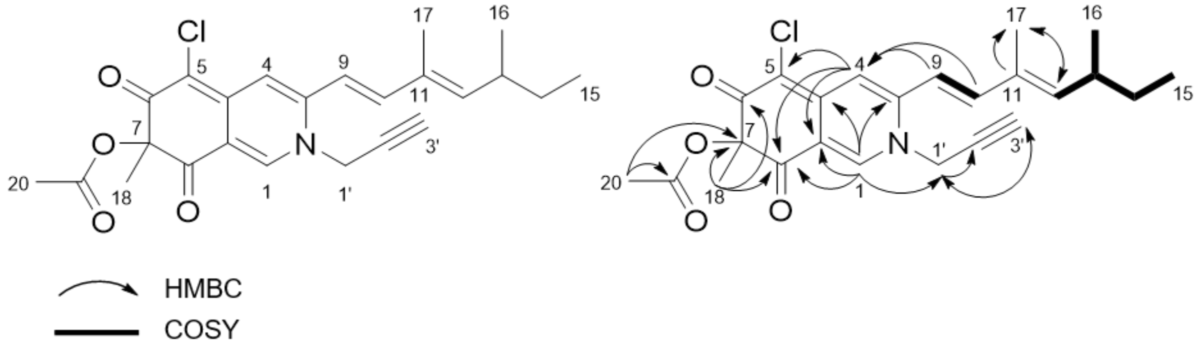

Table S14: ^{1}H NMR spectroscopic data for compounds **3**, **4**, **39′** and **63** recorded at 500 MHz on $CDCl_3$, **24′** recorded at 500 MHz on CD_3CN and **50′** recorded at 500 MHz on MeOD. Compounds **3** and **4** were described on a previous study.[1]

Position	δH (*J* in Hz)					
	**3**	**4**	**24′**	**39′**	**50′**	**63**
1	7.93, s	7.83, s	8.03, s	7.58, s	8.4, s	7.84, s
4	6.86, s	6.99, s	6.85, s	7, s	6.85, s	6.99, s
9	6.13, d(16.1)	6.23, d(15.9)	6.47, d(15.6)	6, d(15.3)	6.46, d(15.4)	6.26, d(15.2)
10	7.04, d(16.7)	6.91, d(15.6)	6.9, d(15.3)	6.94, d(15.4)	6.75, d(15.2)	6.94, d(15.6)
12	5.69, d(9.9)	5.68, d(9.9)	5.65, d(9.1)	5.71, d(10.0)	5.45, d(8.2)	5.70, d(9.7)
13	2.47, m	2.47, m	2.49, m	2.52, m	2.40, m	2.47, m
14	1.40/1.30, m	1.42/1.32, m	1.41/1.29, m	1.47/1.37	1.40/1.30, m	1.43/1.33, m
15	0.84, t(7.5)	0.86, t(7.5)	0.84, m	0.91, t(7.4)	0.87, t(7.3)	0.87, t(7.4)
16	0.99, d(6.6)	1.00, d(6.7)	0.99, d(6.7)	1.05, d(6.9)	0.95, d(6.3)	1.00, d(6.9)
17	1.83, s	1.82, s	1.83, s	1.84, s	1.50, s	1.84, s
18	1.57, s	1.52, s	1.43, s	1.55, s	1.41, s	1.53, s
20	2.16, s	2.13, s	2.2, s	2.19, s	2.14, s	2.14, s
1'		3.99, m	4.69, m	4.17/4.01, s	3.91, m	4.55, d(2.3)
2'		3.91, m		3.07, m		
3'			2.06, m		3.30, m	2.63, t(2.3)
4′			1.27, m	7.34, d(7.2)		
5′			0.84, m	7.13, d(7.4),	5.15, m	
6′			0.84, m	7.30, d(7.2)		
7′				7.13, d(7.4),		
8′				7.34, d(7.2)	7.31, d(7.8)	
9′					7.01, t(7.7)	
10′					7.08, t(7.1)	
11′					7.63, d(7.8)	

Table S15: ^{13}C NMR spectroscopic data for compounds **3**, **4**, **39′** and **63** recorded at 500 MHz on $CDCl_3$, **24′** recorded at 700 MHz on CD_3CN and **50′** recorded at 500 MHz on MeOD. Compounds **3** and **4** were described on a previous study.[1]

Position	δC, type					
	**3**	**4**	**24′**	**39′**	**50′**	**63**
1	138.4, CH	142.0, CH	141.4, CH	141, CH	138.3, CH	140.7, CH
3	146.3, C	144.8, C	151.8, C	144.5, C	147.7, C	144.2, C
4	110.3, CH	111.8, CH	112, CH	111.8, CH	124.9, CH	111.7, CH
4a	147.1, C	148.5, C	145.1, C	147.9, C	148.4, C	147.7, C
5	101.5, C	102.2, C	100.7, C	102.7, C	102.1, C	103.5, C
6	183.7, C	184.4, C	184.2, C	184.6, C	185.4, C	184.9, C
7	85.4, C	84.9, C	86.4, C	85.0, C	86.5, C	85.0, C
8	193.3, C	194.0, C	194.8, C	194, C	195.4, C	193.7, C
8a	114.2, C	114.6, C	116.1, C	114.8, C	117.5, C	115.0, C
9	116.4, CH	115.1, CH	115.1, CH	114.8, CH	112.7, CH	114.6, CH
10	142.9, CH	145.1, CH	146.1, CH	145.1, CH	146.0, CH	145.6, CH
11	132.0, C	131.7, C	133.6, C	131.7, C	133.8, C	132.0, C
12	148.7, CH	148.0, CH	147.2, CH	148.2, CH	147.7, CH	148.6, CH
13	35.1, CH	35.0, CH	35.5, CH	35.2, CH	36.2, CH	35.3, CH
14	30.6, CH_2	30.0, CH_2	30.9, CH_2	30.2, CH_2	31.3, CH_2	30.2, CH_2
15	12.0, CH_3	12.0, CH_3	12.3, CH_3	12.2, CH_3	12.5, CH_3	12.2, CH_3
16	20.1, CH_3	20.2, CH_3	20.6, CH_3	20.5, CH_3	20.7, CH_3	20.4, CH_3
17	12.4, CH_3	12.6, CH_3	13.0, CH_3	12.8, CH_3	12.5, CH_3	12.7, CH_3
18	23.6, CH_3	23.3, CH_3	24.0, CH_3	23.4, CH_3	24.0, CH_3	23.3, CH_3
19	170.9, C	170.2, C	170.7, C	170.2, C	171.7, C	170.4, C
20	20.6, CH_3	20.3, CH_3	20.6, CH_3	20.5, CH_3	20.4, CH_3	20.5, CH_3
1'		55.4, CH_2	42.7, CH	55.6, CH_2	56.6, CH	43.8, CH_2
2'		60.9, CH_2	173.9, C	36.8, CH	174.1, C	75.2, C
3'			30.4, CH_2	136, CH	28.6, CH_2	77.7, CH
4′			25.9, CH	128.8, CH	109.4, C	
5′			22.1, CH_3	129.5, CH	68.9, CH	
6′			23.3, CH_3	127.9, CH		
7′				129.5, CH	ND	
8′				128.8, CH	112.6, CH	
9′					120.6, CH	
10′					122.9, CH	
11′					119.2, CH	
12′					128.3, C	

ND : not determined

(1) Hebra, T.; Elie, N.; Poyer, S.; Van Elslande, E.; Touboul, D.; Eparvier, V. Dereplication, Annotation, and Characterization of 74 Potential Antimicrobial Metabolites from *Penicillium sclerotiorum* Using t-SNE Molecular Networks. *Metabolites* **2021**, *11* (7), 444. DOI: 10.3390/metabo11070444.

(2) Somoza, A. D.; Lee, K.-H.; Chiang, Y.-M.; Oakley, B. R.; Wang, C. C. C. Reengineering an Azaphilone Biosynthesis Pathway in *Aspergillus Nidulans* To Create Lipoxygenase Inhibitors. *Org. Lett.* **2012**, *14* (4), 972–975. DOI: 10.1021/ol203094k.

(3) Eade, R. A.; Page, H.; Robertson, A.; Turner, K.; Whalley, W. B. 986. The Chemistry of Fungi. Part XXVIII. Sclerotiorin and Its Hydrogenation Products. *J. Chem. Soc.* **1957**, 4913. DOI: 10.1039/jr9570004913.

(4) Natsume, M.; Takahashi, Y.; Marumo, S. Chlamydospore-like Cell-Inducing Substances of Fungi: Close Correlation between Chemical Reactivity with Methylamine and Biological Activity. *Agric. Biol. Chem.* **1988**, *52* (2), 307–312. DOI: 10.1271/bbb1961.52.307.

(5) Arai, N.; Shiomi, K.; Tomoda, H.; Tabata, N.; Yang, D. J.; Masuma, R.; Kawakubo, T.; Omura, S. Isochromophilones III -VI, Inhibitors of Acyl-CoA: Cholesterol Acyltransferase Produced by *Penicillium multicolor* FO-3216. *J. Antibiot. (Tokyo)* **1995**, *48* (7), 696–702. DOI: 10.7164/antibiotics.48.696.

(6) Liu, Z.; Qiu, P.; Liu, H.; Li, J.; Shao, C.; Yan, T.; Cao, W.; She, Z. Identification of Anti-Inflammatory Polyketides from the Coral-Derived Fungus *Penicillium* sclerotiorin: In Vitro Approaches and Molecular-Modeling. *Bioorganic Chem.* **2019**, *88*, 102973. DOI: 10.1016/j.bioorg.2019.102973.

(7) Seto, H.; Tanabe, M. Utilization of 13C-13C Coupling in Structural and Biosynthetic Studies. III. Ochrephilone - a New Fungal Metabolite. *Tetrahedron Lett.* **1974**, *15* (8), 651–654. DOI: 10.1016/S0040-4039(01)82295-4.

(8) Matsuzaki, K.; Tahara, H.; Inokoshi, J.; Tanaka, H. New Brominated and Halogen-Less Derivatives and Structure-Activity Relationship of Azaphilones Inhibiting Gp120-CD4 Binding. *J. Antibiot. (Tokyo)* **1998**, *51* (11), 1004–1011. DOI: 10.7164/antibiotics.51.1004.

(9) Fielding, H. C.; Robertson, A.; Travers, R. B.; Whalley, W. B. 371. The Chemistry of Fungi. Part XXXII. The Oxidation of Sclerotioramine and the Structure of Sclerotiorin. *J. Chem. Soc. Resumed* **1957**, 1814–1824. DOI: 10.1039/JR9580001814.

(10) Li, J.; Yang, X.; Lin, Y.; Yuan, J.; Lu, Y.; Zhu, X.; Li, J.; Li, M.; Lin, Y.; He, J.; Liu, L. Meroterpenes and Azaphilones from Marine Mangrove Endophytic Fungus *Penicillium* 303. *Fitoterapia* **2014**, *97*, 241–246. DOI: 10.1016/j.fitote.2014.06.011.

(11) Zhang, L.; Long, Y.; Lei, X.; Xu, J.; Huang, Z.; She, Z.; Lin, Y.; Li, J.; Liu, L. Azaphilones Isolated from an Alga-Derived Fungus *Penicillium* Sp. ZJ-27. *Phytochem. Lett.* **2016**, *18*, 180–186. DOI: 10.1016/j.phytol.2016.10.010.

(12) Kanehisa, M.; Goto, S. KEGG: Kyoto Encyclopedia of Genes and Genomes. *Nucleic Acids Res.* **2000**, *28* (1), 27–30. DOI: 10.1093/nar/28.1.27.
